# YTHDF1 targets the chemotherapy response by suppressing NOTCH1-induced stemness in colorectal cancer

**DOI:** 10.1038/s41392-025-02507-1

**Published:** 2025-12-22

**Authors:** Henley Cheung, Huarong Chen, Danyu Chen, Heming Zhou, Cong Liang, Weixin Liu, Alvin Ho-Kwan Cheung, Yanqiang Ding, Kai Yuan, Xiaoxing Li, Yongxin Zhang, Shiyan Wang, Wei Kang, Ka-Fai To, Housheng He, Chi Chun Wong, Jun Yu

**Affiliations:** 1 Institute of Digestive Disease and Department of Medicine and Therapeutics, State Key Laboratory of Digestive Disease, Li Ka Shing Institute of Health Sciences, CUHK-Shenzhen Research Institute, The Chinese University of Hong Kong, Hong Kong SAR, China; 2 Department of Anesthesia and Intensive Care, The Chinese University of Hong Kong, Hong Kong SAR, China; 3 Department of Anatomical and Cellular Pathology, The Chinese University of Hong Kong, Hong Kong SAR, China; 4 Institute of Precision Medicine, The First Affiliated Hospital, Sun Yat-sen University, Guangzhou, China; 5 Princess Margaret Cancer Centre, University Health Network, Toronto, ON Canada

**Keywords:** Cancer stem cells, Cancer therapy

## Abstract

N^6^-methyladenosine (m^6^A) modification of mRNAs is a predominant epigenetic regulatory mechanism in tumor initiation and progression. Cancer stem cells (CSCs) are the key drivers of colorectal cancer (CRC) initiation and chemotherapy resistance. Here, we found that the m^6^A reader YT521-B homologous domain family, member 1 (YTHDF1), promotes CRC stemness, tumorigenesis, and chemotherapy resistance. YTHDF1 protein expression was positively correlated with CD133 and LGR5 expression in human CRC tissues (N = 184, *P* < 0.001 for both markers). YTHDF1 promoted m^6^A-dependent self-renewal in CSCs and patient-derived organoids and increased the tumor-initiating potential in vivo. Lgr5-specific *Ythdf1*-KI mice presented accelerated *Apc*^*Min/+*^ (*P* < 0.05) and AOM/DSS (*P* < 0.05)-induced colorectal tumorigenesis, whereas Lgr5-specific *Ythdf1* knockout in *Apc*^*Min/+*^ mice inhibited tumorigenesis (*P* < 0.01). Integrative multiomic profiling revealed NOTCH1 as a downstream target. YTHDF1 binds m^6^A-modified *NOTCH1*, promoting its translation and enhancing NOTCH signaling. NOTCH1 knockdown or blockade by the γ-secretase inhibitor DAPT abolished YTHDF1-mediated tumorigenesis in *Ythdf1* knock-in mice (*P* < 0.01). YTHDF1 promoted resistance to oxaliplatin and 5-fluorouracil in CSCs by inhibiting apoptosis and DNA damage. AOM/DSS-treated *Ythdf1* knock-in mice presented increased resistance to oxaliplatin (*P* < 0.001) and 5-fluorouracil (*P* < 0.05). Translationally, in vivo targeting of YTHDF1 via VNP-encapsulated si*YTHDF1* or salvianolic acid C inhibited tumor growth (*P* < 0.05 for both treatments) and increased treatment efficacy when VNP was combined with oxaliplatin (*P* < 0.05, SAC: *P* < 0.01) or 5-fluorouracil (*P* < 0.05 for both treatments). In conclusion, YTHDF1 promotes stemness and chemoresistance in CRC via NOTCH1 activation. Targeting YTHDF1 is a promising strategy to improve the outcome of chemotherapy in CRC.

## Introduction

Colorectal cancer (CRC) is the third most common and second deadliest cancer in the world,^1^ highlighting the urgent need for novel therapeutic strategies. Despite recent advances in early detection and clinical intervention, approximately 38.5% of patients with stage II-III CRC relapse after primary tumor resection and adjuvant chemotherapy, contributing to a poor 5-year survival rate of 6.5%.^2^ The current treatments for localized CRC involve surgery, radiotherapy, together with adjuvant chemotherapy to eliminate potential remaining cancer cells in the body, with the folinic acid-fluorouracil-oxaliplatin (FOLFOX) regimen as a therapeutic cornerstone.^3^ For metastatic CRC, chemotherapies such as FOLFOX are common first-line therapies. These chemotherapeutic agents target highly proliferative tumor cells by inducing DNA damage and triggering cell death. However, a small subpopulation of cancer stem cells (CSCs), endowed with self-renewal properties reminiscent of normal stem cells, frequently evade these therapies, driving chemoresistance and sustaining tumor relapse.^4^ In CRC, CSCs are commonly identified by the expression of gene markers, such as CD133,^5^ CD44,^6^ and LGR5.^7^ With 40–50% of stage II-III CRC patients exhibiting chemotherapy resistance and relapse, and the poor response of metastatic CRC patients,^8^ overcoming these challenges remains a critical priority.^9^ Targeting the molecular mechanisms involved in sustaining colorectal CSCs is a promising strategy to improve treatment efficacy and patient outcomes.

Although the comprehensive efforts by the Cancer Genome Atlas (TCGA) and the International Cancer Genome Consortium (ICGC) have identified key somatic mutations and transcriptional dysregulation during CRC development, posttranscriptional mechanisms, such as the modification of RNA molecules, that contribute to tumorigenesis remain poorly understood. Among >100 types of RNA modifications, RNA N^6^-methyladenosine (m^6^A) is the most prominent in humans, and it regulates mRNA splicing, translation, and degradation. m^6^A eukaryotes, is dynamically and tightly modulated by “writers” (METTL3, METTL14, WTAP) that catalyze the formation of m^6^A in mRNA, “erasers” (ALKBH5, FTO) that catalyze the demethylation of m^6^A-modified nucleotides, and “readers” (YT521-B homologous (YTH) domain family,^10^ Insulin-like growth factor 2 mRNA-binding protein family^11^) that recognize m^6^A-modified transcripts. Key m^6^A readers from the YTH domain family^10^ and the insulin-like growth factor 2 mRNA-binding protein family, in turn, determine mRNA fate by regulating mRNA stability, translation, and splicing. Emerging evidence highlights the critical role of m^6^A modifications in colorectal tumorigenesis.^12–14^ For instance, METTL3 has been demonstrated to facilitate CRC through activating mTOR signaling.^12^ YTHDF1 also accelerates CRC development by promoting ARHGEF2 translation and RhoA signaling.^13^ Whereas ALKBH5 upregulation in CRC cells recruits myeloid-derived suppressor cells (MDSCs) to suppress immune surveillance, leading to disease progression.^14^ These studies illustrate the multifaceted roles of m^6^A modification to CRC tumorigenesis.

Among m^6^A readers, YTHDF1, a YTH domain protein, drives the translation of m^6^A-modified mRNAs by interacting with translation initiation factors.^15^ Notably, YTHDF1 stands out as the top upregulated m^6^A regulator in CRC, with studies linking it to tumor growth, metastasis, and the modulation of antitumor immunity in a m^6^A-dependent manner.^13,16^ Recent reports have also implied that YTHDF1 promotes cancer stemness, tumorigenesis, and chemoresistance.^17–19^ Nevertheless, the CSC-specific role of m^6^A modification in CRC remains underexplored. Most of the published studies focusing on YTHDF1 rely on the use of CRC cell lines, which cannot recapitulate the cellular hierarchy and stemness-differentiation continuum of the in vivo tumors. Furthermore, it is unclear whether genetic or pharmacological interventions targeting YTHDF1 could overcome YTHDF1-associated cancer stemness and chemoresistance.

In this study, we hypothesize that the m^6^A reader YTHDF1 is critically involved in colorectal CSCs, thereby triggering resistance to chemotherapeutic agents. Multi-cohort analysis revealed that YTHDF1 expression in CRC positively correlated with CSC markers LGR5 and CD133, inferring a positive functional role of YTHDF1 in colorectal CSCs. To better mimic the role of CSCs in tumorigenesis and chemoresistance, we evaluated the oncogenic role of YTHDF1 by using Lgr5-specific *Ythdf1* knock-in and knockout mouse models of CRC, leading to specific manipulation of YTHDF1 in LGR5-expressing lineage. Consistent with our hypothesis, *Ythdf1* knock-in mice demonstrated profound resistance to oxaliplatin (OXA) and 5-fluorouracil (5-FU) compared to wild-type mice. Integrative multiomic profiling demonstrated that NOTCH1 is a critical downstream target of YTHDF1 that promotes cancer stemness and chemoresistance. YTHDF1 binds to m^6^A-modified NOTCH1 mRNA to promote its translation. Therapeutically, targeting of YTHDF1 in vitro and in vivo with vesicle-like nanoparticle (VNP)-encapsulated si*YTHDF1* or YTHDF1 selective inhibitor salicylic acid C (SAC) suppressed tumor growth and synergistically enhanced OXA and 5-FU treatment efficacy in vivo. This represents the first comprehensive evaluation of the role and clinical significance of YTHDF1 in colorectal CSCs and associated chemoresistance, and our findings position YTHDF1 as a promising therapeutic target to overcome chemoresistance associated with CSCs and improve treatment outcomes in CRC.

## Results

### YTHDF1 promotes self-renewal capacity in a m^6^A-dependent manner

**Fig. 1:**
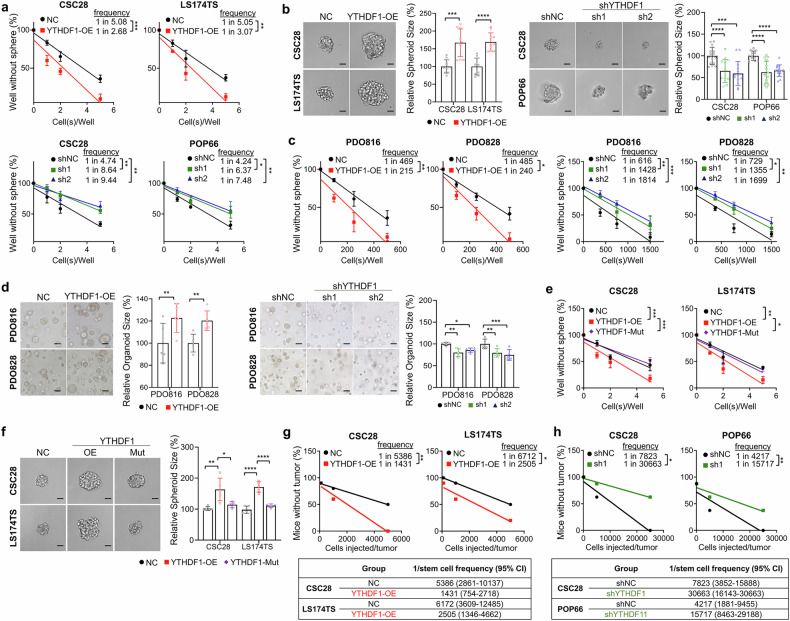
YTHDF1 promotes self-renewal capacity in a m^6^A-dependent manner. **a** Effects of YTHDF1 overexpression and knockdown on CSC28, LS174TS, and POP66 self-renewal capacity (seeded at 1, 2, 5, and 10 cells per well, 7 days) and **b** spheroid formation ability (N = 10 in the overexpression group, N = 15 in the knockdown group). Scale bar = 20 μm. **c** Effects of YTHDF1 overexpression and knockdown on the self-renewal capacity and **d** organoid formation ability of PDO816 and PDO828 (N = 5 per group). Scale bar = 40 μm. **e** Effects of YTHDF1 overexpression and the YTHDF1 mutation on the self-renewal capacity of CSC28 and LS174TS cells (seeded at 1, 2, 5, and 10 cells per well for 7 days). **f** Spheroid formation ability (n = 5 per group). Scale bar = 20 μm. **g** In vivo limiting dilution of CSC28 and LS174TS cells with YTHDF1 overexpression, injected with 100, 1000, 5000, and 25,000 cells with 1/stem cell frequency (95% CI) analysis. **h** In vivo limiting dilution of CSC28 and POP66 with shYTHDF1 injected with 100, 5000, 25,000, and 50,000 cells with 1/stem cell frequency (95% CI) analysis. All the experiments were conducted in biological triplicate. Spheroid and organoid size quantifications were performed via ImageJ. The data are presented as the means ± SDs. *p < 0.05; **p < 0.01; ***p < 0.001; ****p < 0.0001; ELDA extreme limiting dilution analysis (**a**, **c**, **e**, **g**, **h**), 2-tailed t-test (**b**, **d**), one-way ANOVA (**b**, **d**, **f**)

To elucidate the functional role of YTHDF1 in stemness, we utilized three-dimensional CSC cultures and CRC patient-derived organoids (PDOs) to recapitulate tumor physiology.^20,21^ In CSC28 and LS174TS cells, YTHDF1 overexpression enhanced self-renewal capacity (Fig. 1a), spheroid formation (Fig. 1b), and cell viability (Supplementary Fig. 1a). Similarly, YTHDF1 overexpression in PDO816 and PDO828 enhanced self-renewal capacity (Fig. 1c) and organoid formation (Fig. 1d). In contrast, YTHDF1 knockdown had opposite effects on CSCs (Fig. 1a, b; Supplementary Fig. 1a) and PDOs (Fig. 1c, d). To determine whether the stemness modulation by YTHDF1 is m^6^A-dependent, we overexpressed either wild-type YTHDF1 or m^6^A-binding-deficient mutant YTHDF1 (K395A, Y397A) in CSC28 and LS174TS cells. Western blotting confirmed the successful overexpression of wild-type and mutant YTHDF1 (Supplementary Fig. 1b). Consistent with the role of YTHDF1 as a m^6^A reader, the effects of YTHDF1 on promoting self-renewal capacity (Fig. 1e), spheroid formation (Fig. 1f), and cell viability (Supplementary Fig. 1c) were abolished in mutant YTHDF1, underscoring that the effect of YTHDF1 on stemness is exerted through m^6^A interactions. To confirm the role of YTHDF1 in CRC stemness in vivo, we performed limiting dilution assays by subcutaneously injecting varying doses of CSCs with either YTHDF1 overexpression or knockdown into NSG mice. YTHDF1 overexpression in CSC28 and LS174TS cells increased the tumor-initiating potential and increased the stem cell frequency compared with those of the controls (Fig. 1g, Supplementary Fig. 1d). In contrast, CSC28 and POP66 cells with YTHDF1 knockdown had impaired tumor-initiating potential and reduced stem cell frequency (Fig. 1h, Supplementary Fig. 1d). Collectively, our findings indicate that YTHDF1 promotes stemness properties in colorectal CSCs and PDOs in a m^6^A-dependent manner.

### Lgr5-specific *Ythdf1* knock-in in mice accelerates colorectal tumorigenesis

**Fig. 2:**
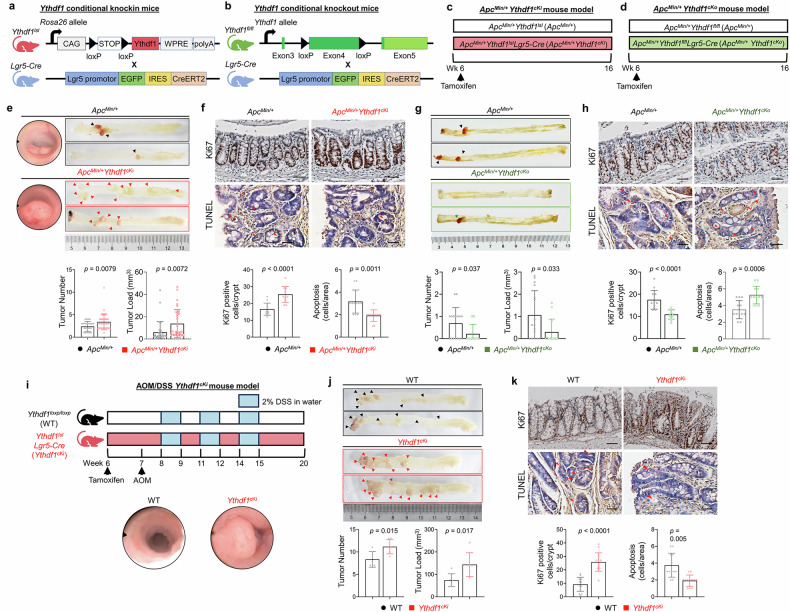
Lgr5-specific *Ythdf1* conditional knock-in in mice accelerates colorectal tumorigenesis. **a** Illustration of *Ythdf1*^*lsl*^*Lgr5-Cre* mouse construction. **b** Illustration of *Ythdf1*^*fl/fl*^*Lgr5-Cre* mouse construction. **c** Experimental schematic of *Ythdf1*^*lsl*^*Lgr5-Cre* in the *Apc*^*Min/+*^ induced CRC model. **d** Experimental schematic of *Ythdf1*^*fl/fl*^*Lgr5-Cre* in the *Apc*^*Min/+*^ induced CRC model. **e** Representative images of colonoscopy (*top left*). Representative images of the colon (*top* *right*). Tumor number and load in *Apc*^*Min/+*^(N = 31) and *Apc*^*Min/+*^*Ythdf1*^*cKi*^ (N = 35) mice (*bottom*). **f** Ki67 and TUNEL staining of colon tissues from *Apc*^*Min/+*^ (n = 12) and *Apc*^*Min/+*^*Ythdf1*^*cKi*^ (n = 12) mice; scale bar = 25 μm (*top*). Statistical analysis of Ki67-positive cells/crypt- and TUNEL-positive cells (*bottom*). **g** Representative images of the colon (*top*). Tumor number and load in *Apc*^*Min/+*^ (N = 16) and *Apc*^*Min/+*^*Ythdf1*^*cKo*^ (N = 14) mice (*bottom*). **h** Ki67 and TUNEL staining of colon tissues from *Apc*^*Min/+*^ (n = 12) and *Apc*^*Min/+*^*Ythdf1*^*cKo*^ (n = 12) mice; scale bar, 25 μm (*top*). Statistical analysis of Ki67-positive cells/crypt- and TUNEL-positive cells (*bottom*). **i** Experimental schematic of *Ythdf1*^*lsl*^*Lgr5-Cre* in the AOM/DSS^*-*^induced CRC model (*top*). Representative images of colonoscopy (*bottom*). **j** Representative images of the colon (*top*). Tumor number and load in WT (N = 6) and *Ythdf1*^*cKi*^ (N = 6) mice (*bottom*). **k** Ki67 and TUNEL staining of colon tissues from littermate WT (n = 15) and *Ythdf1*^*cKi*^ (n = 15) mice (*top*). Statistical analysis of Ki67-positive cells/crypt- and TUNEL-positive cells (*bottom*). Scale bar = 25 μm. The data are presented as the means ± SDs. *p < 0.05; **p < 0.01; ***p < 0.001; ****p < 0.0001; 2-tailed t-test (**e**, **f**, **g**, **h**, **j**, **k**)

To further investigate the role of YTHDF1 in promoting CRC in colon stem cells, the full-length coding sequence of *Ythdf1* was inserted into the *Rosa26* locus of C57BL/6 mice. These mice were then bred with *Lgr5-Cre*^*ERT2*^ mice to establish Lgr5-specific *Ythdf1* knock-in mice (*Ythdf1*^*lsl*^*Lgr5-Cre*^*ERT2*^, termed *Ythdf1*^*cKi*^) (Fig. 2a). On the other hand, we targeted exon 4 of *Ythdf1*, encoding the YTH domain, which flanks loxP sites, and crossed these mice with *Lgr5-Cre*^*ERT2*^ mice to generate Lgr5-specific *Ythdf1* knockout mice (*Ythdf1*^*fl/fl*^*Lgr5*-*Cre*^*ERT2*^, termed *Ythdf1*^*cKo*^) (Fig. 2b). To assess whether Lgr5-specific manipulation of YTHDF1 modulates spontaneous colorectal tumorigenesis, we crossbred these mouse lines with *Apc*^*Min/+*^ mice to generate *Apc*^*Min/+*^*Ythdf1*^*lsl*^*Lgr5-Cre*^*ERT2*^ (*Apc*^*Min/+*^*Ythdf1*^*cKi*^) and *Apc*^*Min/+*^*Ythdf1*^*flfl*^*Lgr5-Cre*^*ERT2*^ (*Apc*^*Min/+*^*Ythdf1*^*cKo*^) mice. The mice were administered tamoxifen (100 mg/kg) for 5 consecutive days to activate *Cre* recombinase and were harvested after 16 weeks (Fig. 2c, d). Compared with *Apc*^*Min/+*^ littermates, *Apc*^*Min/+*^*Ythdf1*^*cKi*^ mice presented greater tumor numbers (*P* = 0.0079) and tumor loads (*P* = 0.0072) (Fig. 2e). This effect was accompanied by increased proliferation marker Ki67^+^ cells per crypt in the colon (*P* < 0.0001) and inhibition of apoptosis (*P* = 0.0011), as indicated by terminal deoxynucleotidyl transferase-mediated deoxyuridine triphosphate nick-end labeling (TUNEL) staining (Fig. 2f). In contrast, *Apc*^*Min/+*^*Ythdf1*^*cKo*^ mice presented significant reductions in tumor number (*P* = 0.037) and tumor load (*P* = 0.033) (Fig. 2g), with reduced cell proliferation (*P* < 0.0001) and enhanced apoptosis (*P* = 0.0006), as shown by Ki67 and TUNEL staining, respectively (Fig. 2h).

We independently validated the protumorigenic function of YTHDF1 in an azoxymethane/dextran sodium sulfate (AOM/DSS)-induced CRC model. *Ythdf1*^*cki*^ mice were administered tamoxifen (100 mg/kg) for 5 consecutive days to activate *Cre* recombinase, followed by AOM injection and three DSS cycles (Fig. 2i). Consistent with the *Apc*^*Min/+*^ model, *Ythdf1*^*cKi*^ exacerbated colorectal tumorigenesis in terms of both tumor number (*P* = 0.015) and tumor load (*P* = 0.017) (Fig. 2j), with increased cell proliferation (*P* < 0.0001) and suppressed apoptosis (*P* = 0.0005), as determined by Ki67 and TUNEL staining (Fig. 2k). These findings collectively offer evidence that Lgr5-specific YTHDF1 knock-in in mice promotes colorectal tumorigenesis.

### Multiomic profiling identifies NOTCH1 as a downstream target of YTHDF1

**Fig. 3:**
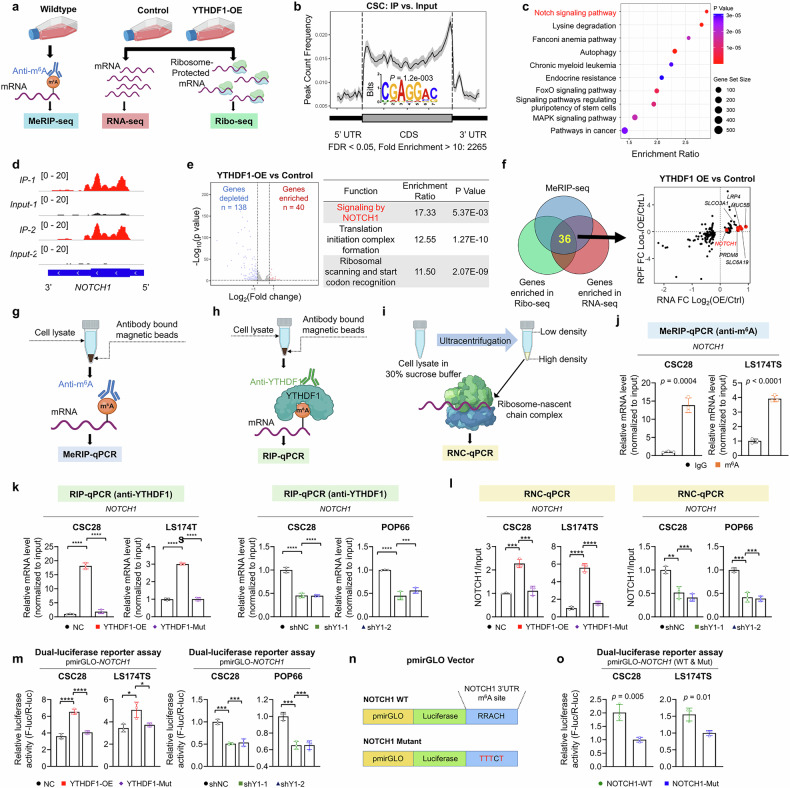
Multiomic profiling identified NOTCH1 as a downstream target of YTHDF1. **a** Schematic of integrative MeRIP-seq, RNA-seq, and Ribo-seq. **b** MeRIP-seq of CSC28 revealed the normalized distribution of m^6^A peaks and identified m^6^A motifs. **c** Pathway enrichment analysis (GSEA-KEGG) of the top enriched genes among the MeRIP-seq data. **d** The m^6^A peak of NOTCH1 was located at the 3′ UTR. **e** RNA-seq revealed that NOTCH1 signaling was the most enriched pathway in YTHDF1-overexpressing CSC28 cells. **f** Schematic of overlapping MeRIP-seq, RNA-seq, and Ribo-seq. **g** Schematic of MeRIP-qPCR. **h** Schematic of RIP-qPCR. **i** Schematic of RNC-qPCR. **j** Level of m^6^A modification on *NOTCH1* mRNA (N = 3 per group). **k** Levels of *NOTCH1* mRNA in YTHDF1-overexpressing and YTHDF1-mutant CSCs (N = 3 per group). **l** Levels of *NOTCH1* mRNA in the active RNC complex (N = 3 per group). **m** Relative *NOTCH1* luciferase activity in YTHDF1 wild-type or mutant-overexpressing or YTHDF1-knockdown CSCs (N = 3 per group). **n** pmirGLO-NOTCH1-mutant reporters with mutated m^6^A sites (RRACH to TTTCT) in the 3′UTR. **o** Relative luciferase activities of the pmirGLO-NOTCH1 or pmirGLO-NOTCH1-mutant reporter in CSCs (N = 3 per group). The data are presented as the means ± SDs. *p < 0.05; **p < 0.01; ***p < 0.001; ****p < 0.0001; 2-tailed t-test (**j**, **o**), ordinary one-way ANOVA (**k**, **l**, **m**)

Given that YTHDF1-driven CRC stemness critically depends on m^6^A, we performed methylated RNA immunoprecipitation sequencing (MeRIP-seq) in wild-type CSC28 cells, as well as RNA sequencing (RNA-seq) and ribosome sequencing (Ribo-Seq) in control and YTHDF1-overexpressing CSC28 cells (Fig. 3a), to determine the underlying mechanism. The m^6^A consensus motif GGAC was enriched in wild-type CSC28, with m^6^A peaks predominantly found in the coding sequence and 3′UTR (Fig. 3b). Intriguingly, the Notch signaling pathway, which is known for regulating stem cell differentiation and the DNA damage response, exhibited the greatest m^6^A enrichment in colorectal CSCs (Fig. 3c). Consistent with previous findings, *NOTCH1* mRNA exhibited significant m^6^A modifications at the 3’ UTR (Fig. 3d).^22^ RNA-seq revealed 138 downregulated and 40 upregulated transcripts in the YTHDF1-overexpressing CSC28 cells, with gene ontology (GO) analysis highlighting NOTCH1 signaling among the most upregulated transcripts (Fig. 3e). Integration of Ribo-seq data, which focused on transcripts with increased translation in YTHDF1-overexpressing cells, narrowed the list down to 36 potential YTHDF1 downstream targets, with NOTCH1 emerging as the top candidate (Fig. 3f). TMA cohort analysis (cohort I, N = 184) further revealed a positive correlation between YTHDF1 and the NOTCH1 protein (χ^2^ = 14.44; *P* = 0.006) in tumor tissues but not in paired normal tissues (Supplementary Fig. 2a). Western blot analysis of patient CRC tissues and paired adjacent normal tissues confirmed the upregulation of both YTHDF1 and NOTCH1 (Supplementary Fig. 2b). These findings indicate that NOTCH1 is a potential YTHDF1 target in colorectal CSCs.

To verify the relationships between YTHDF1 and NOTCH1, we performed MeRIP-qPCR (Fig. 3g), RNA immunoprecipitation (RIP)-qPCR (Fig. 3h), and ribosome-nascent chain complex (RNC)-qPCR (Fig. 3i) to investigate the m^6^A level of *NOTCH1* mRNA, its interaction with YTHDF1, and whether YTHDF1 promotes *NOTCH1* translation, respectively. We first successfully pulled down *NOTCH1* mRNA with an anti-m^6^A antibody, confirming that it is indeed m^6^A modified in CSC28 and LS174TS (Fig. 3j). We then demonstrated the direct interaction between the YTHDF1 protein and m^6^A-modified *NOTCH1* mRNA, as shown by the detection of *NOTCH1* mRNA after the pulldown of YTHDF1 in both CSC28 and LS174TS cells (Supplementary Fig. 2c). Furthermore, wild-type YTHDF1 overexpression in CSCs significantly increased immunoprecipitated *NOTCH1* mRNA levels (Fig. 3k), whereas the m^6^A-binding-deficient mutant YTHDF1 (K395A, Y397A) had no effect. Conversely, YTHDF1 knockdown significantly reduced NOTCH1 mRNA levels, supporting a m^6^A-dependent interaction. Wild-type YTHDF1 overexpression, but not mutant YTHDF1 overexpression, increased ribosome-associated *NOTCH1* mRNA in CSC28 and LS174TS cells (Fig. 3l), whereas YTHDF1 knockdown reduced ribosome-associated *NOTCH1* mRNA. Additionally, we constructed a dual-luciferase reporter plasmid containing the 3′UTR of *NOTCH1*. Overexpression of wild-type YTHDF1 significantly increased luciferase reporter activity, whereas mutant YTHDF1 had no equivalent effects. YTHDF1 knockdown reduced luciferase reporter activity, suggesting that YTHDF1 m^6^A-dependently upregulates *NOTCH1* mRNA translation (Fig. 3m). Finally, a luciferase reporter with mutated m^6^A motifs in the NOTCH1 3′UTR (Fig. 3n) exhibited diminished luciferase reporter activity compared with its wild-type counterpart in CSC28 and LS174TS cells (Fig. 3o). Taken together, these results demonstrate that YTHDF1 promotes *NOTCH1* mRNA translation through direct binding to its m^6^A-modified 3′UTR.

### NOTCH1 is a functional target of YTHDF1 in promoting stemness and tumorigenesis

**Fig. 4:**
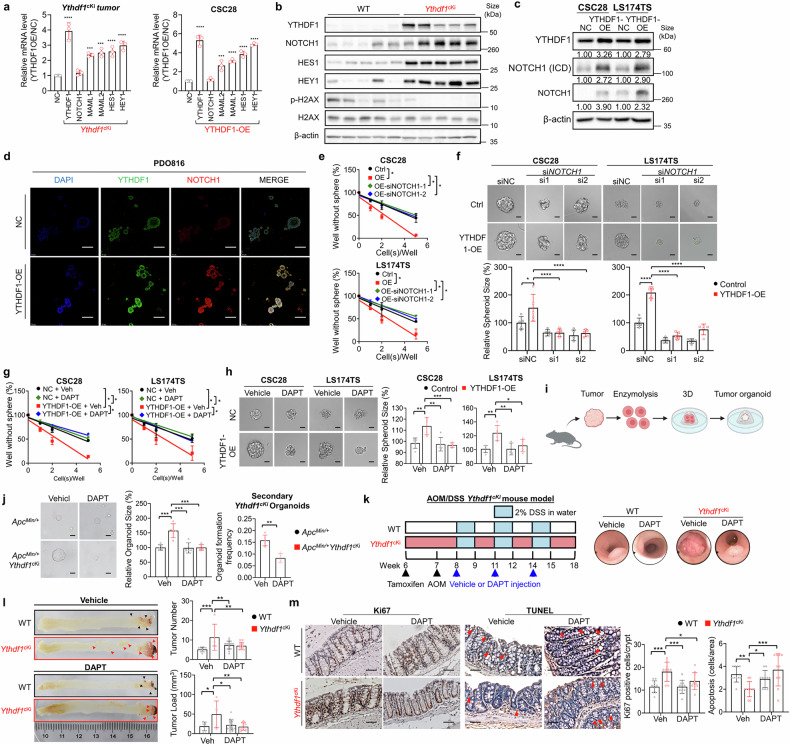
NOTCH1 is a functional target of YTHDF1 in promoting stemness and tumorigenesis. **a** *Ythdf1* conditional knock-in activated the NOTCH signaling pathway at the mRNA level (N = 3 per group). **b** NOTCH1, HES1, HEY1, p-H2AX, and H2AX protein expression in the *Ythdf1* conditional knock-in mouse model. **c** NOTCH1 protein expression in YTHDF1-overexpressing CSCs. **d** Representative images of immunofluorescence co-staining of YTHDF1 and NOTCH1 in control and YTHDF1-overexpressing PDO816 cells. Scale bar = 100 μm. **e** Effect of siNOTCH1 in the context of YTHDF1 overexpression on CSC28 and LS174TS self-renewal capacity (seeded at 1, 2, 5, and 10 cells per well for 7 days) and **f** spheroid formation ability (n = 5 per group). Scale bar = 20 μm. **g** Effect of DAPT in YTHDF1-overexpressing cells on CSC28 and LS174TS self-renewal capacity (seeded at 1, 2, 5, and 10 cells per well, 7 days) and **h** spheroid formation ability (n = 5 per group). Scale bar = 20 μm. **i** Schematic of intestinal tumor organoid construction. **j** Effect of DAPT on *Apc*^*Min/+*^ and *Apc*^*Min/+*^*Ythdf1*^cKi^ organoid formation (*left and middle*) and secondary organoid formation ability (*right*) (N = 5 per group). Scale bar = 20 μm. **k** Experimental schematic of *Ythdf1*^*lsl*^*Lgr5-Cre* in the AOM/DSS^*-*^induced CRC model (*left*). Representative images of colonoscopy (*right*). **l** Representative images of the colon (*left*). Tumor number and load in WT (N = 7) and *Ythdf1*^*cKi*^ (N = 5) mice and in DAPT-treated WT (N = 17) and *Ythdf1*^*cKi*^ (N = 11) mice (*right*). **m** Ki67 staining of colon tissues from littermate WT (n = 12) and *Ythdf1*^*cKi*^ (n = 12) mice. Scale bar = 25 μm. Spheroid and organoid size quantifications were performed via ImageJ. The data are presented as the means ± SDs. *p < 0.05; **p < 0.01; ***p < 0.001; ****p < 0.0001; ordinary one-way ANOVA (**a**); two-way ANOVA (**f**, **h**, **j**, **l**, **m**); ELDA: extreme limiting dilution analysis (**e**, **g**)

Given the crucial role of Notch signaling in maintaining cancer stemness,^23,24^ we investigated whether YTHDF1-induced NOTCH1 translation drives stemness in colorectal CSCs. Using tumor samples collected from the Lgr5-specific *Ythdf1*^*cki*^ mouse model, qPCR analysis revealed that YTHDF1 knock-in increased the expression of NOTCH1 downstream targets, including MAML1, MAML2, HES1, and HEY1 (Fig. 4a). Similar results were observed in CSC28. The upregulated expression of these targets was confirmed at the protein level via Western blot analysis of the tumor samples (Fig. 4b). Notably, NOTCH1 protein expression was elevated without a corresponding increase in *NOTCH1* mRNA, further demonstrating the translation-promoting role of YTHDF1. NOTCH1 is known to negatively regulate the DNA damage response, with phosphorylated H2AX (p-H2AX) serving as a marker of DNA damage and therapy resistance in CRC.^25,26^ In *Ythdf1*^*cki*^ tumors, p-H2AX levels were lower than those in wild-type tumors, indicating reduced DNA damage. Consistent with YTHDF1-mediated NOTCH1 translation, both full-length NOTCH1 and its active intracellular domain were upregulated in YTHDF1-overexpressing CSC28 and LS174TS cells (Fig. 4c, Supplementary Fig. 3a). Conversely, YTHDF1 depletion diminished NOTCH1 protein expression (Supplementary Fig. 3b). Immunofluorescence in PDO816 further confirmed NOTCH1 upregulation upon YTHDF1 overexpression (Fig. 4d). To test functional dependency, we silenced NOTCH1 with siRNA in YTHDF1-overexpressing CSC28 and LS174TS cells, which abolished the YTHDF1-driven enhancements in self-renewal capacity (Fig. 4e), spheroid formation (Fig. 4f), and cell viability (Supplementary Fig. 3c) but had no effect on the controls. Similarly, YTHDF1-overexpressing CSCs treated with the NOTCH inhibitor DAPT showed abrogation of YTHDF1-induced self-renewal capacity (Fig. 4g), spheroid formation (Fig. 4h), and cell viability (Supplementary Fig. 3d). Furthermore, we constructed intestinal tumor organoids derived from the colon tumors of *Apc*^*Min/+*^ and *Apc*^*Min/+*^*Ythdf1*^*cKi*^ mice (Fig. 4i). DAPT treatment selectively suppressed the growth of the *Apc*^*Min/+*^*Ythdf1*^*cKi*^ organoids but not the *Apc*^*Min/+*^ organoids (Fig. 4j). Importantly, 10 µM DAPT suppressed the capacity of dissociated *Apc*^*Min/+*^*Ythdf1*^*cKi*^ organoids to initiate secondary organoid formation, indicating a disruption of tumor-initiating functions (Fig. 4j).

To validate the role of NOTCH1 in CRC, we administered DAPT to *Ythdf1*^*cki*^ mice with AOM/DSS-induced colorectal tumorigenesis (Fig. 4k). DAPT treatment significantly reduced the tumor number (*P* = 0.0064) and tumor burden (*P* = 0.004) in *Ythdf1*^*cki*^ mice (Fig. 4l) but had no significant effect on wild-type mice. Notably, DAPT treatment nullified the difference in tumorigenesis between *Ythdf1*^*cki*^ and its wild-type counterparts. Ki67 and TUNEL staining revealed suppressed cell proliferation (*P* = 0.01) and increased apoptosis (*P* = 0.0002) specifically in DAPT-treated *Ythdf1*^*cki*^ mice compared with vehicle-treated *Ythdf1*^*cki*^ mice (Fig. 4m). Collectively, these data establish NOTCH1 as a critical downstream effector of YTHDF1, driving stemness and tumorigenesis in CRC through translational regulation.

### YTHDF1 overexpression promotes chemoresistance in CRC

**Fig. 5:**
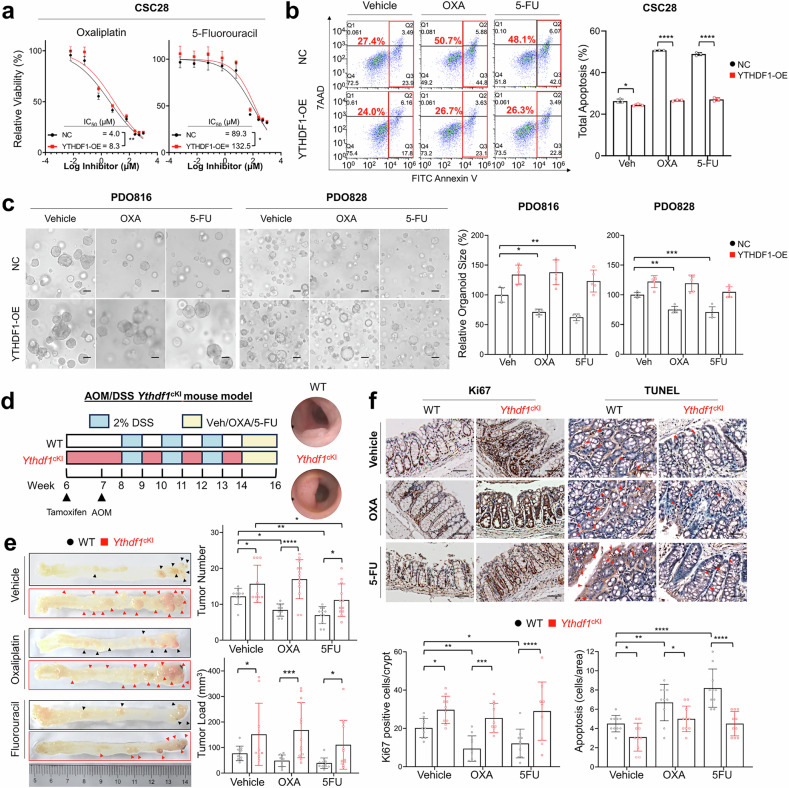
YTHDF1 overexpression promotes chemoresistance in CRC. **a** IC_50_ values of YTHDF1-overexpressing CSC28 cells with increased resistance to oxaliplatin and 5-fluorouracil. **b** Apoptosis assay of YTHDF1-overexpressing CSC28 cells treated with oxaliplatin and 5-fluorouracil (n = 5 per group). **c** Organoid formation assay in YTHDF1-overexpressing PDOs treated with oxaliplatin and 5-fluorouracil (n = 5 per group). Scale bar = 20 μm. **d** Experimental schematic of *Ythdf1*^*lsl*^*Lgr5-Cre* in the AOM/DSS-induced CRC model (*left*). Representative images of colonoscopy (*right*). **e** Representative images of the colon, tumor number, and load in WT mice treated with vehicle (n = 10), OXA (n = 10), or 5-FU (n = 19). *Ythdf1* knock-in mice were treated with vehicle (n = 10) or OXA (n = 13), or 5-FU (n = 12). **f** Ki67 staining of colon tissues from the AOM/DSS model (n = 10 for each group). Organoid size was quantified via ImageJ. The data are presented as the means ± SDs. *p < 0.05; **p < 0.01; ***p < 0.001; ****p < 0.0001; nonlinear regression (**a**); two-way ANOVA (**b**, **c**, **e**, **f**)

Multicellular tumor spheroids enriched with CSCs exhibit increased resistance to drug-induced apoptosis, a defining feature of chemoresistance.^27^ To explore the translational relevance of YTHDF1 in the CRC chemotherapy response, we investigated the effect of YTHDF1 manipulation on sensitivity to two first-line chemotherapeutic agents used in CRC, OXA and 5-FU. In vitro IC_50_ assays demonstrated that CSC28 and LS174TS cells overexpressing YTHDF1 exhibited increased chemoresistance to OXA and 5-FU, as evidenced by increased IC_50_ values (Fig. 5a and Supplementary Fig. 4a). CSC28 cells overexpressing YTHDF1 also displayed resistance to OXA- and 5-FU-induced apoptosis, as confirmed by an Annexin V^+^ assay (Fig. 5b). PDOs are considered a reliable preclinical model for the assessment of drug response.^28^ While OXA and 5-FU effectively suppressed the expansion of PDO816 and PDO828, the same chemotherapeutic agents failed to reduce the size of the organoids overexpressing YTHDF1 (Fig. 5c), highlighting the role of YTHDF1 in driving chemoresistance.

We further investigated the function of YTHDF1 in promoting chemoresistance in vivo. Lgr5-specific *Ythdf1*^*cki*^ mice were subjected to an AOM/DSS-induced CRC model. Following tumor confirmation by colonoscopy, the mice were treated with vehicle, OXA, or 5-FU (Fig. 5d). Notably, OXA treatment in *Ythdf1*^*cki*^ mice did not reduce the tumor number or tumor load (Fig. 5e), whereas the same OXA regimen significantly reduced the tumor number (P < 0.0001) and tumor load (P = 0.0008) in wild-type mice. Similarly, *Ythdf1*^*cki*^ mice displayed resistance to 5-FU, whereas 5-FU suppressed tumor number (P = .015) and tumor load (P = .0037) in wild-type mice (Fig. 5e). Histological analysis revealed that OXA or 5-FU treatment in *Ythdf1*^*cki*^ mice neither inhibited tumor cell proliferation nor induced apoptosis (Fig. 5f), which is consistent with the chemoresistance phenotypes. In contrast, OXA- or 5-FU-treated wild-type mice demonstrated a significant reduction in tumor cell proliferation and increased apoptosis (Fig. 5f). These results highlight YTHDF1 as a critical mediator of chemoresistance in vivo.

### YTHDF1 knockdown overcomes chemoresistance in CRC

**Fig. 6:**
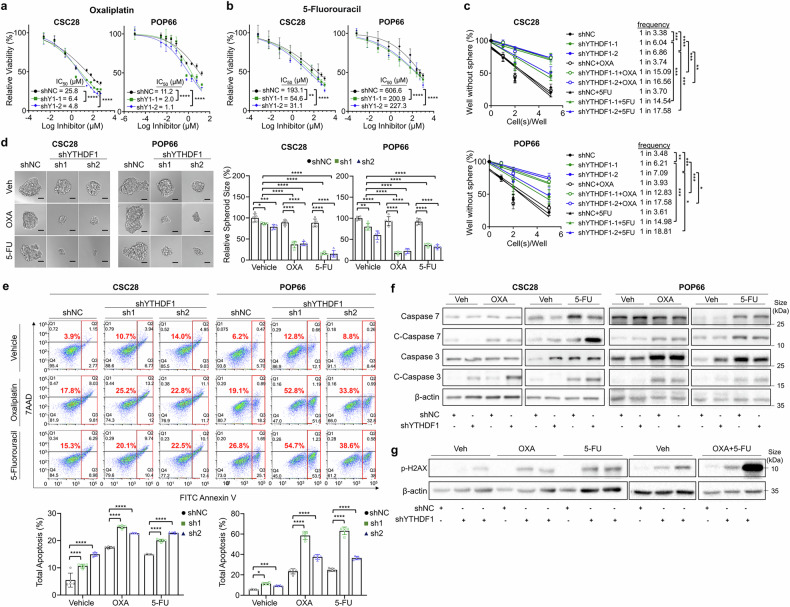
YTHDF1 knockdown overcomes chemoresistance in CRC. **a** and **b** IC_50_ values of oxaliplatin and 5-fluorouracil in CSCs and impaired chemoresistance induced by shYTHDF1. **c** Effects of oxaliplatin and 5-fluorouracil on the self-renewal capacity of YTHDF1-knockdown CSCs (seeded at 1, 2, 5, and 10 cells per well for 7 days) and **d** spheroid formation ability (n = 5 per group). Scale bar = 20 μm. **e** Apoptosis assay of YTHDF1-knockdown CSC28 and POP66 cells treated with oxaliplatin and 5-fluorouracil (n = 5 per group). **f** Expression of apoptosis markers in YTHDF1-knockdown CSCs treated with oxaliplatin and 5-fluorouracil. **g** Expression of DNA damage markers in CSC28 with YTHDF1 knockdown treated with oxaliplatin and 5-fluorouracil. The data are presented as the means ± SDs. *p < 0.05; **p < 0.01; ***p < 0.001; ****p < 0.0001; nonlinear regression (**a**, **b**); ELDA extreme limiting dilution analysis (**c**); two-way ANOVA (**d**, **e**)

We then asked whether YTHDF1 depletion could reverse chemoresistance in colorectal CSCs. Indeed, in vitro IC_50_ assays revealed that compared with the control, YTHDF1 knockdown sensitized CSC28 and POP66 to OXA and 5-FU, as evidenced by decreased IC_50_ values (Fig. 6a, b), reduced self-renewal capacity (Fig. 6c), spheroid formation (Fig. 6d), and increased apoptosis (Fig. 6e). Western blot analysis of key apoptosis markers validated these observations, revealing elevated expression of cleaved caspase-3 and cleaved caspase-7 in YTHDF1-knockdown CSCs treated with OXA or 5-FU (Fig. 6f, Supplementary Fig. 4b). Notably, p-H2AX levels in CSC28 cells with YTHDF1 knockdown were significantly enriched after treatment with OXA, 5-FU, or their combination, whereas total H2AX levels were unchanged (Fig. 6g, Supplementary Fig. 4b, c). These results suggest that loss of YTHDF1 enhances chemotherapy-induced DNA damage and apoptotic signaling in colorectal CSCs, thereby lowering their chemoresistance capacity.

### Targeting YTHDF1 via VNP-si*YTHDF1* or salvianolic acid C enhances the efficacy of chemotherapy

**Fig. 7:**
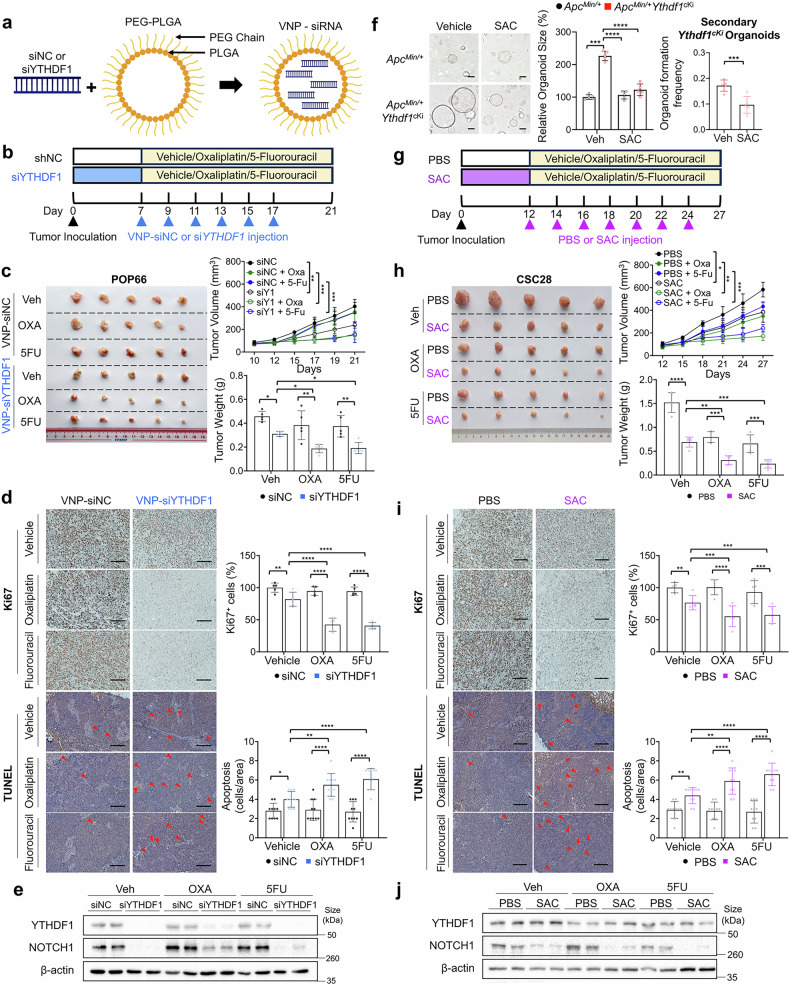
Targeting YTHDF1 via VNP-si*YTHDF1* or salvianolic acid C enhances the efficacy of chemotherapy. **a** Structure of vesicle-like nanoparticles encapsulating si*YTHDF1*. **b** Experimental schematic of the POP66 xenograft model (n = 5 for each group). **c** Effect of VNP-si*YTHDF1* on POP66 xenografts with or without chemotherapy treatment in the context of YTHDF1 knockdown (N = 5 per group). **d** Ki67 and TUNEL staining of tumor tissue with or without chemotherapy treatment in the context of YTHDF1 knockdown (N = 10 per group). **e** Protein expression of YTHDF1 and NOTCH1 in tumor tissues. **f** Effects of SAC on *Apc*^*Min/+*^ and *ApcMin*^*/+*^*Ythdf1*^*cKi*^ organoid formation (*left and middle*) and secondary organoid formation ability (*right*) (N = 5 per group). Scale bar = 20 μm. **g** Experimental schematic of the xenograft model with CSC28 cells (n = 5 for each group). **h** Effect of SAC on POP66 xenografts with or without chemical treatment in the context of YTHDF1 knockdown (N = 5 per group). **i** Ki67 and TUNEL staining of tumor tissue with or without chemotherapy treatment in the context of YTHDF1 knockdown (N = 10 per group). **j** Protein expression of YTHDF1 and NOTCH1 in tumor tissues. Organoid size was quantified via ImageJ. The data are presented as the means ± SDs. *p < 0.05; **p < 0.01; ***p < 0.001; ****p < 0.0001; two-way ANOVA (**c**, **d**, **h**, **i**)

Next, we investigated whether targeting YTHDF1 via nanoparticle-delivered siRNA or pharmacological agents could enhance the efficacy of chemotherapy. We employed PLGA-based vesicle-like nanoparticles (VNPs) encapsulating *YTHDF1*-siRNA for targeted silencing (Fig. 7a).^29^ Prior studies have shown that VNP-si*YTHDF1* is safe and well-tolerated in mice.^16^ NSG mice bearing established CSC28 xenografts were randomized and treated with either VNP-siNC or VNP-si*YTHDF1* alone or in combination with OXA or 5-FU (Fig. 7b). Compared with VNP-siNC, VNP-si*YTHDF1* reduced tumor volume (P = 0.015) and weight (P = 0.037) (Fig. 7c). Importantly, more substantial tumor growth inhibition was observed in the VNP-si*YTHDF1* plus OXA (tumor volume: P = 0.022, tumor weight: P = 0.023) or VNP-si*YTHDF1* plus 5-FU (tumor volume: P = 0.048, tumor weight: P = 0.0047) groups than in the VNP-siNC plus OXA or VNP-siNC plus 5-FU groups (Fig. 7c). VNP-si*YTHDF1* in combination with OXA or 5-FU also synergistically reduced proliferation and increased apoptosis in tumors compared with those in all other groups (Fig. 7d). Western blotting confirmed successful YTHDF1 knockdown and consequent NOTCH1 downregulation in tumor tissues following VNP-si*YTHDF1* treatment (Fig. 7e). Safety assessments revealed no significant changes in body weight, histology or serum markers of liver (alanine aminotransferase, aspartate transaminase) or kidney function (creatinine, blood urea nitrogen) in mice treated with VNP-siNC or VNP-siYTHDF1 (Supplementary Fig. 4d, e), confirming the tolerability of VNP-based therapy. These findings demonstrate that targeting YTHDF1 with VNP-siYTHDF1 is a safe and effective strategy to increase chemotherapy efficacy.

Furthermore, we investigated the effect of salvianolic acid C (SAC), a natural selective YTHDF1 inhibitor.^30^ In vitro studies revealed that SAC suppressed the growth of tumor organoids derived from *Apc*^*Min/+*^*Ythdf1*^*cKi*^ mice but had no effect on *Apc*^*Min/+*^ tumor organoids (Fig. 7f). SAC (10 µM) suppressed secondary organoid formation in *Apc*^*Min/+*^*Ythdf1*^*cKi*^ tumor organoids, indicating the disruption of tumor-initiating functions (Fig. 7f). SAC effectively reversed the effects of YTHDF1 overexpression on cell viability (Supplementary Fig. 4f) and spheroid formation (Supplementary Fig. 4g) in CSC28 and LS174TS cells. In vitro IC_50_ assays revealed that SAC treatment sensitized CSC28 and POP66 cells to OXA and 5-FU, as evidenced by increased IC_50_ values (Supplementary Fig. 4h). Treatment of the CSC xenografts with SAC, chemotherapy (OXA or 5-FU), or their combination (Fig. 7g) demonstrated that SAC alone suppressed tumor growth (tumor volume: P = 0.036; tumor weight: P < 0.0001) (Fig. 7h). Compared with SAC alone, the combination of SAC with OXA further enhanced tumor suppression (tumor volume: P < 0.05; tumor weight: P < 0.01) or OXA (tumor volume: P < 0.01; tumor weight: P < 0.001) (Fig. 7h). Similar additive effects were observed in the SAC plus 5-FU group compared with the SAC (tumor volume: P < 0.05; tumor weight: P < 0.001) or 5-FU (tumor volume: P < 0.001; tumor weight: P < 0.001) groups (Fig. 7h). Ki67 and TUNEL staining confirmed that, compared with single agent treatment, SAC combined with OXA or 5-FU had greater anti-proliferative and pro-apoptotic effects (Fig. 7i). Western blot analysis confirmed that SAC suppressed YTHDF1 and downstream NOTCH1 expression in tumor tissues (Fig. 7j).

### YTHDF1 is positively correlated with CSC markers in CRC patient cohorts

**Fig. 8:**
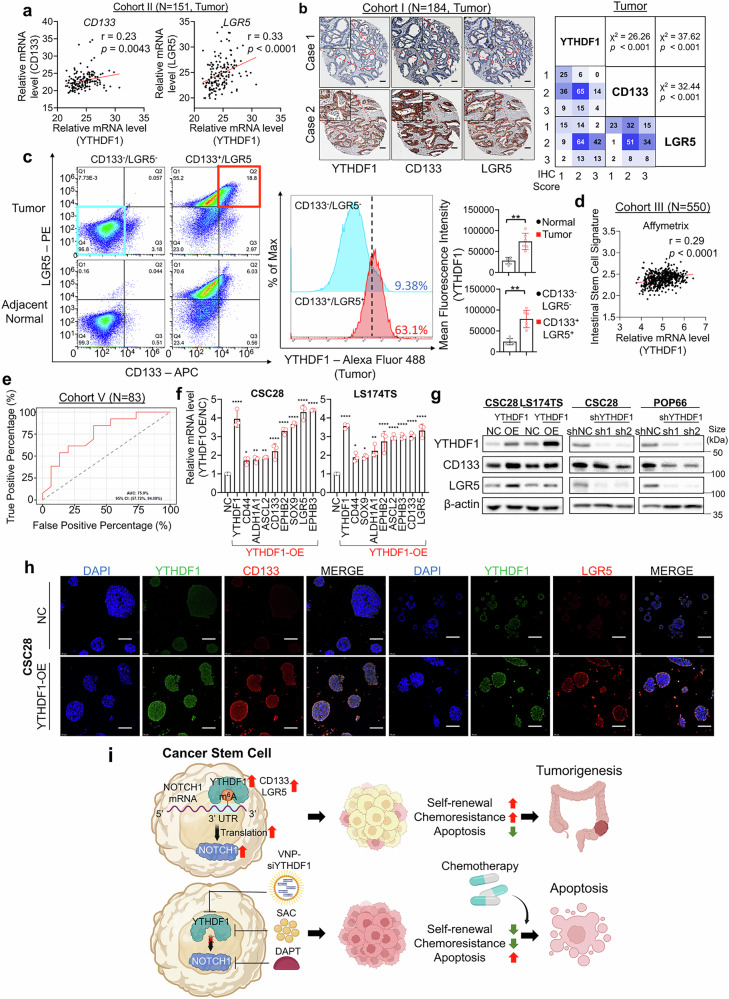
YTHDF1 is positively correlated with CSC markers in CRC patient cohorts. **a** *YTHDF1* mRNA is positively correlated with *CD133* (*left*) and *LGR5* (*right*). **b** Representative images of YTHDF1, CD133, and LGR5 staining in CRC tissue microarrays (N = 184) (*left*). Scale bar = 100 μm. Pearson correlation analysis of YTHDF1, CD133, and LGR5 protein expression (*right*). **c** YTHDF1 is enriched in the CD133^+^/LGR5^+^ double-positive CSC population isolated from fresh CRC tumors and adjacent normal tissues. **d** YTHDF1 expression is positively correlated with the LGR5 stem cell signature. **e** ROC curves for YTHDF1 and the response of CRC patients to chemotherapy. **f** Overexpression of YTHDF1 is coupled to the induction of stemness marker expression at the mRNA level (N = 3 per group). **g** Overexpression of YTHDF1 promoted CD133 and LGR5 expression. **h** Representative images of immunofluorescence co-staining of YTHDF1, CD133, and LGR5 in control and YTHDF1-overexpressing cells. Scale bar = 100 μm. **i** Schematic diagram showing the mechanism of YTHDF1-induced chemoresistance in CSCs. Figure created with BioRender.com. The data are presented as the means ± SDs. *p < 0.05; **p < 0.01; ***p < 0.001; ****p < 0.0001; Pearson correlation (**a**, **d**), Pearson χ^2^ test (**b**); 2-tailed t-test (**c**); ordinary one-way ANOVA (**f**)

Finally, we evaluated YTHDF1 mRNA expression in our in-house CRC patient cohort (cohort II, N = 151), revealing that YTHDF1 was positively correlated with two established colorectal CSC markers, CD133 (r = 0.23, P = 0.0043) and LGR5 (r = 0.33, P < 0.0001) (Fig. 8a), in tumor tissues, whereas the housekeeping gene ACTB was not correlated with either gene (Supplementary Fig. 5a). At the protein level, YTHDF1 expression in TMA Cohort I was positively correlated with CD133 (χ^2^ = 26.26; *P* < 0.001) and LGR5 (χ^2^ = 37.62; *P* < 0.001) (Fig. 8b) expression in CRC tissues, whereas CD133 and LGR5 expression was also positively correlated (χ^2^ = 32.44; *P* < 0.001). In paired adjacent normal tissues in the same TMA cohort, YTHDF1 expression was weakly correlated with CD133 (χ^2^ = 8.31; P < 0.01) and LGR5 (χ^2^ = 11.25; P < 0.001), whereas CD133 and LGR5 expression was not correlated (χ^2^ = 2.16; P > 0.05) (Supplementary Fig. 5b). In Cohort IV, which included paired CRC tumor and adjacent normal tissues (N = 12), we consistently found positive correlations between the protein expression of YTHDF1 and that of CD133 and LGR5 (Supplementary Fig. 5c). TCGA cohort analysis (cohort III, N = 550) also validated a positive correlation between *YTHDF1* and *LGR5* mRNA expression (*P* < 0.0001) in CRC (Supplementary Fig. 5d). In addition, the YTHDF1 and LGR5 proteins were consistently upregulated in CRC tumor tissues compared with paired adjacent normal tissues in the TCGA database (Supplementary Fig. 5e). Analysis of a CRC patient-derived single-cell RNA sequencing dataset (GSE132465) confirmed the positive correlations among YTHDF1, CD133, and LGR5 expression (Supplementary Fig. 5f).

To validate these findings, we collected 6 pairs of fresh CRC tissues and adjacent normal tissues. Using a magnetic bead-based separation system, we isolated double-negative (CD133^-^/LGR5^-^) and double-positive (CD133^+^/LGR5^+^) cell populations. The CD133^+^/LGR5^+^ cancer stem cell population was significantly enriched in tumor tissues compared with adjacent normal tissues (Supplementary Fig. 5g). Furthermore, YTHDF1 expression was significantly elevated in CD133^+^/LGR5^+^ tumor cells relative to that in CD133^-^/LGR5^-^ tumor cells (Fig. 8c).

Next, an LGR5 stem cell signature was derived via two independent microarray platforms, Affymetrix and Agilent.^31^ YTHDF1 expression in Cohort III was positively correlated with the full LGR5 stem cell expression signature across both platforms, suggesting that YTHDF1 expression is correlated with cancer stemness in human patients (Fig. 8d, Supplementary Fig. 5h). Furthermore, random forest machine learning analysis of a public CRC patient dataset (cohort V, N = 83) with chemotherapy responders and nonresponders revealed that elevated YTHDF1 expression was associated with chemotherapy nonresponders (AUC = 75.9%, 95% CI: 57.72%–94.08%) (Fig. 8e). This finding was corroborated in another public CRC patient dataset (cohort VI, N = 68) (Supplementary Fig. 5i). Taken together, analyses of independent cohorts inferred to potential associations between YTHDF1, stemness, and chemoresistance in CRC.

Given the association between YTHDF1 and CSC markers in CRC patients, we investigated whether YTHDF1 modulates stemness features in vitro. YTHDF1 overexpression in the colorectal CSCs CSC28 and LS174TS upregulated the mRNA expression of CD133 and LGR5, alongside other stemness-associated markers, including *ALDH1A1*, *ASCL2*, *CD44*, *EPHB2*, *EPHB3*, and *SOX9* (Fig. 8f). This effect was mirrored by elevated CD133 and LGR5 protein expression, as determined by Western blotting (Fig. 8g, Supplementary Fig. 5j) and immunofluorescence staining (Fig. 8h), whereas YTHDF1 knockdown had the opposite effect. YTHDF1 overexpression also decreased the protein expression of KRT20, a marker of colonocyte differentiation (Supplementary Fig. 5k).

Together, our study identified YTHDF1 as a key modulator of CRC stemness and chemoresistance. Targeting YTHDF1 or its downstream effector NOTCH1 mitigates chemoresistance in CRC, and combining YTHDF1 inhibition with chemotherapy represents a promising strategy to improve treatment efficacy (Fig. 8i).

## Discussion

A number of reports have suggested that YTHDF1 plays a functional role in cancer stemness and chemoresistance; however, these studies are based on cancer cell lines, thus lacking the in vivo context of an evolving tumor comprising a mixed stem cell-like and more differentiated cell populations. To address this issue, we employed comprehensive transgenic mouse models with Lgr5-expressing, colon stem cell lineage-specific knock-in and knockout of YTHDF1, and demonstrated that CSC-specific YTHDF1 manipulation in mice drives CRC tumorigenesis and elicits chemotherapy resistance. The functional importance of YTHDF1 in CSCs was further validated in CSC cell lines and CRC organoids. Integrative multiomic profiling revealed that YTHDF1 promotes chemoresistance through direct binding to m^6^A-modified *NOTCH1* mRNA. Loss or inhibition of YTHDF1 disrupted *NOTCH1* translation, induced DNA damage, and thus synergized with commonly used chemotherapies in CRC, 5-FU and Oxaliplatin, to potentiate tumor eradication in vivo. Targeting YTHDF1 with nanoparticle-delivered siRNA or a selective pharmacological inhibitor promoted chemosensitivity in xenograft tumor models established from colorectal CSCs. Our combination approach of YTHDF1 inhibition plus chemotherapies synergistically suppressed tumor growth, highlighting its potential for CRC treatment.

The significance of YTHDF1 lies in its specificity for CSCs, which are increasingly recognized as the primary mediators of tumor initiation, metastasis, and relapse in CRC. A series of in vitro and in vivo studies demonstrated the CSC-specific role of YTHDF1 in promoting tumorigenesis and chemoresistance. These results align with emerging evidence that m^6^A modifications can dynamically regulate CSC phenotypes across cancer types. In this connection, the potential function of YTHDF1 in CRC stemness is supported by its positive correlation with stemness markers CD133 and LGR5 across multiple CRC patient cohorts, as well as with LGR5 stem cell expression signatures and chemotherapy nonresponders. We also confirmed that YTHDF1 promoted CD133 and LGR5 expression in CSCs, xenograft models, and CSC-specific *Ythdf1* knock-in/knockout mice, suggesting that YTHDF1 directly boosts cancer stemness properties. This stemness modulation is tightly coupled to m^6^A modifications, as evidenced by the lack of CSC-promoting effect of YTHDF1 mutants lacking the functional m^6^A-binding region. The robust phenotypes in *Ythdf1*^*cki*^ mice and *Ythdf1*^*cko*^, in modulating tumorigenesis, whilst having no apparent effects on normal colon functionality, offer compelling functional evidence of the cancer stemness-modulating role of YTHDF1 in CRC. Our findings confirm the protumorigenic role of YTHDF1 in CRC^13^ and show that YTHDF1 overexpression only in the colon stem cell compartment is sufficient to generate protumorigenic and chemoresistant phenotypes. This specificity implies that YTHDF1-targeted therapies could selectively disrupt CSC self-renewal without broadly affecting normal stem cells in the colon, a critical consideration for minimizing toxicity in CRC treatment. m^6^A dependency of YTHDF1 also raises intriguing questions about its interplay with other m^6^A writers and erasers. While our study focused on YTHDF1, the broader m^6^A regulatory network likely contributes to CSC phenotypes, highlighting the need for a systems-level understanding of m^6^A regulation to fully elucidate its definitive role in colorectal CSCs.

Our mechanistic dissection involving integrative MeRIP-seq, RNA-seq, and Ribo-seq analyses identified NOTCH1 as a top colorectal CSC-specific target of YTHDF1. NOTCH1 expression was positively correlated with YTHDF1 in CRC patient cohorts. YTHDF1 binds directly to the m^6^A-modified 3’UTR of *NOTCH1*, increasing its translation. Elevated NOTCH1 expression and NOTCH signaling activation were observed in YTHDF1-overexpressing colorectal CSCs, CRC organoids, and tumors from *Ythdf1*^*cki*^ mice. *NOTCH1* mRNA was abundantly detected in the ribosome-nascent chain complex in YTHDF1-overexpressing CSCs but not in YTHDF1-mutant CSCs, confirming m^6^A-dependent regulation. Hence, YTHDF1 promotes the ribosome loading of m^6^A-modified *NOTCH1* and facilitates its protein translation. NOTCH1 inhibition with siRNA or the γ-secretase inhibitor DAPT reversed YTHDF1-driven CSC self-renewal in vitro. DAPT treatment in *Ythdf1*^*cki*^ mice attenuated YTHDF1-induced CRC formation. These results, which are consistent with the established role of Notch signaling in CRC stemness^32–37^ and adenoma formation,^38^ define a m^6^A-YTHDF1-NOTCH1 axis with therapeutic potential.

We further demonstrated that YTHDF1 and downstream NOTCH signaling are key regulators of chemoresistance in CRC. Ectopic YTHDF1 expression reduced DNA damage, as shown by decreased p-H2AX levels in tumors from *Ythdf1*^*cki*^ mice. This effect stems from YTHDF1 promoting the translation of NOTCH1, which in turn modulates the DNA damage response.^26^ YTHDF1 overexpression in colorectal CSCs and primary CRC organoids provoked resistance to OXA and 5-FU. Compared with their wild-type counterparts, *Ythdf1*^*cki*^ mice were remarkably resilient to OXA and 5-FU; in particular, OXA was largely ineffective. Given the critical role of YTHDF1 in promoting CRC stemness and chemoresistance, we explored its translational potential in CSCs. YTHDF1 depletion exacerbated DNA damage and sensitized CSCs and PDOs to OXA- and 5-FU-induced proliferative arrest and apoptosis. VNP-si*YTHDF1* or the YTHDF1 inhibitor SAC suppressed NOTCH1 signaling, increasing the efficacy of OXA and 5-FU against colorectal CSCs. Collectively, our findings suggest that YTHDF1 is a potential therapeutic target that can overcome chemoresistance in CRC, a major bottleneck in CRC management.^2,4^

Our work also indicates that targeting YTHDF1 might be a viable strategy for blocking aberrant NOTCH signaling in cancer. Although several NOTCH inhibitors have been developed, such as γ-secretase and transcriptional complex inhibitors, they broadly target NOTCH1-4 signaling, often causing gastrointestinal toxicity due to nonspecific disruption, thereby limiting potential utility in the clinic.^39–41^ As a consequence, thus far, no FDA approval has been given for NOTCH inhibitors for CRC treatment. Nonspecific NOTCH inhibitors may disrupt NOTCH2, which functions as a tumor suppressor in CRC,^42–44^ potentially promoting tumorigenesis. Alternatively, targeting YTHDF1 offers a selective approach to suppress NOTCH1 activity while minimizing toxicity compared with pan-NOTCH inhibition, given that mRNAs of other NOTCH isoforms are not targeted by YTHDF1 in our analysis. Our nanoparticle-encapsulated YTHDF1-siRNA appears safe and effective, with no reported changes in body weight or liver/kidney functions, supporting our notion that targeting YTHDF1 will provide superior safety and selectivity over pan-NOTCH inhibitors.

In conclusion, our study revealed that the m^6^A reader YTHDF1 drives the m^6^A-YTHDF1-NOTCH1 axis in colorectal CSCs, promoting NOTCH1 translation to increase stemness and chemoresistance in vitro and in vivo. The genetic or pharmacological blockade of YTHDF1 effectively overcomes chemoresistance and suppresses CRC progression when combined with chemotherapeutic agents, highlighting YTHDF1 as a compelling therapeutic target for CRC.

## Materials and methods

### Ethics approval and consent to participate

Informed consent was obtained from all patients, and this study was approved by the Human Ethics Committee of the Chinese University of Hong Kong (Ref. No. 2019.425) and Sun Yat-sen University (Ref. No. [2023] 639). All animal experiments in this study were approved by the Animal Experimentation Ethics Committee (Ref: 22-082-MIS) of CUHK and Xiamen University.

### Human CRC cohorts

Cohort I was obtained from Beijing University Cancer Hospital and included 184 CRC patients. Tissue microarrays (TMAs) were established via the use of paraffinized tumor blocks from this cohort. The staining scores were determined by pathologists. Cohort II consisted of 151 paired adjacent normal and CRC tissues collected from the Prince of Wales Hospital, The Chinese University of Hong Kong, with histologically confirmed CRC. The samples were snap-frozen in liquid nitrogen and stored at –80 °C until RNA extraction. Cohort III, sourced from the TCGA COAD dataset acquired via Xenabrowser (https://xenabrowser.net/), comprised 85 adjacent normal and 465 CRC tissues. Cohort IV consisted of 12 protein samples of paired adjacent normal and CRC tissues collected at the Prince of Wales Hospital in Hong Kong. Cohort V was derived from a public dataset (GSE28702) comprising 83 CRC cases (58 of 83 samples were selected for the training set, and the remaining 25 samples were selected as the test set). Cohort VI was obtained from a public dataset (GSE72968) consisting of 68 CRC cases (40 of 68 samples were selected for the training set, and the remaining 28 samples were selected as the test set). The clinicopathological features of the CRC cohorts are provided in Supplementary Tables 1–3, and the antibodies used for TMA are provided in Supplementary Table 4. Informed consent was obtained from all patients, and this study was approved by the Human Ethics Committee of the Chinese University of Hong Kong (Ref. No. 2019.425) and Sun Yat-sen University (Ref. No. [2023] 639).

### Transgenic mouse models with Lgr5-specific *Ythdf1* knock-in and knockout

Conditional *Ythdf1* knock-in and knockout C57BL/6 mice were generated by the Shanghai Model Organisms Center (Shanghai, China) and crossed with *LGR5-Cre*^*ERT2*^ mice to establish Lgr5-specific *Ythdf1* knock-in mice (*Ythdf1*^*lsl*^LGR5-Cre) and knockout mice (*Ythdf1*^fl/fl^LGR5-Cre). For the *Apc*^*Min/+*^CRC mouse model, *Ythdf1*^*lsl*^LGR5-cre and *Ythdf1*^fl/fl^LGR5-cre mice were crossbred with *Apc*^*Min/+*^ mice to generate *Apc*^*Min/+*^*Ythdf1*^*lsl*^LGR5-cre and *Apc*^*Min/+*^*Ythdf1*^fl/fl^LGR5-cre mice. Tamoxifen (100 mg/kg, Sigma‒Aldrich #T5648) was intraperitoneally injected at week 6.

In the carcinogen-induced CRC mouse model, *Ythdf1*^*lsl*^LGR5-cre mice received an intraperitoneal injection of tamoxifen (100 mg/kg) at week 6 and AOM (10 mg/kg, Sigma‒Aldrich #A5486) at week 7. One week post-injection, the mice were given water containing 2% (wt/vol) DSS (MP Biomedicals #9011-18-1) for 7 days, followed by access to regular drinking water. The DSS treatment was repeated for two more cycles. After the DSS treatment was complete, oxaliplatin (7.5 mg/kg) was administered intraperitoneally once a week for 3 weeks, and 5-fluorouracil (50 mg/kg) was administered intraperitoneally twice a week for 3 weeks. All animal experiments in this study were approved by the Animal Experimentation Ethics Committee (Ref: 22-082-MIS) of CUHK and Xiamen University.

Maximum tumor size/burden permitted: A single tumor must not exceed 20 mm at the largest diameter in an adult mouse. The tumor volume must not exceed 2000 mm^3^. The maximal tumor size/burden was not exceeded in this study.

### Cell lines and organoid models derived from colorectal cancer patients

Human cancer stem cells (CSC28, LS174TS, POP66) and CRC patient-derived organoids (PDO816 and PDO828) were kindly provided by Professor Catherine O’Brien at Princess Margaret Cancer Center, University of Toronto. The HEK293T cell line was purchased from Invitrogen. Mouse organoids were established from fresh colon tumor tissue from *Apc*^*Min/+*^ mice and *Apc*^*Min/+*^*Ythdf1*^*lsl*^LGR5-cre mice. All the cell lines were recently authenticated via STR profiling.

### Limiting dilution assay (LDA)

For in vitro LDA, the number of disassociated CSCs was counted via a hemocytometer, and the cells were serially diluted to 10, 5, 2, and 1 cells/well and then dispensed into sterile 96-well plates with the following distribution: 12 wells for 10 cells, 24 wells for 5 and 2 cells, and 60 wells for 1 cell. The number of disassociated organoids was counted via a hemocytometer, and the organoids were serially diluted to 1000, 500, 250, and 100 cells/well and then dispensed into sterile 96-well plates with the following distributions: 6 wells for 1000 cells, 12 wells for 500 and 250 cells, and 30 wells for 100 cells. The experiments were performed in biological triplicate. After 1–2 weeks, the spheroids or organoids in the wells were scored via a microscope, and the tumor initiation frequency was calculated via ELDA software. For the in vivo LDA, the cells were serially diluted to achieve 25,000, 5000, 1000, and 100 cells/injection for the YTHDF1-overexpressing group and 50,000, 25,000, 5000, and 100 for the YTHDF1-knockdown group. Tumor formation was monitored for 4–8 weeks (up to 6 months for low/no tumor formation), and the results were analyzed via ELDA software to determine the initiating frequency and statistical significance.

### Secondary organoid plating assay

Primary organoids from control and drug-treated wells were dissociated via TrypLE™ Express Enzyme (1X, Thermo Fisher) and replated at a density of 50 cells/µL. The secondary organoids were cultured for 14 days in organoid medium without further drug treatment.^45,46^

### Quantification of spheroid and organoid size

The growth of the colorectal cancer spheroids and organoids was captured via an inverted light microscope. The largest z-plane at which spheroids had a sharp boundary was selected for imaging. The surface area of the organoids in each random field was measured via ImageJ.

### Vesicle-like nanoparticle formulation

The VNP nanoparticles were assembled by Guangzhou Kelan Biotechnology Co., Ltd. (Guangzhou, China). 2′-O-Methyl-modified siRNAs were purchased from GenePharma Co., Ltd. (Shanghai, China). The sequences of the human YTHDF1 siRNAs used are as follows: sense (CCACUCAAACUCUUUCGGGTT) and antisense (CCCGAAAGAGUUUGAGUGGAA).

## Supplementary information

Supplementary File

Western blot raw data

Raw data for rebuttal

## Data Availability

All the data required to support the conclusions of this study are provided in the main text and Supplementary Materials. The raw sequence data reported in this paper have been deposited in the Genome Sequence Archive (accession number: CRA031512) and can be accessed at: https://ngdc.cncb.ac.cn/biosample/browse/SAMC5997392.
