# Supplementary material for: YTHDF1 targets the chemotherapy response by suppressing NOTCH1-induced stemness in colorectal cancer
Source: Signal Transduct Target Ther. 2025 Dec 22;10:409. doi: 10.1038/s41392-025-02507-1 (PMC12719390; doi:10.1038/s41392-025-02507-1)
Supplement: Supplementary file 1 — Supplementary File [file 41392_2025_2507_MOESM1_ESM.docx]

**Supplementary Materials**

Targeting YTHDF1 Enhances Chemotherapy Response by Suppressing NOTCH1 Induced Stemness in Colorectal Cancer

Henley Cheung^1^, Huarong Chen^1,2^, Danyu Chen^1^, Heming Zhou^1^, Cong Liang^1^, Weixin Liu^1^, Alvin Ho-Kwan Cheung^3^, Yanqiang Ding^1^, Kai Yuan^1^, Xiaoxing Li^4^, Yongxin Zhang^4^, Shiyan Wang^4^, Wei Kang^3^, Ka-Fai To^3^, Housheng Hansen He^5^, Chi Chun Wong^1^*, Jun Yu^1^*

Correspondence to: [junyu@cuhk.edu.hk](mailto:junyu@cuhk.edu.hk)

**This PDF file includes:**

Supplementary Materials and Methods

Figures. S1 to S5

Tables S1 to S6

Materials and Methods

**Subcutaneous Xenograft Mouse Models**

For subcutaneous *in vivo* limiting dilution, CSC28 and LS174TS were subcutaneously injected into the left and right flanks of 4-week-old male NSG mice. We injected 100, 1000, 5000, and 25000 YTHDF1-overexpressing cells or 100, 5000, 25000, and 50000 cells YTHDF1-knockdown cells. Tumor size was measured every 2 days with a digital caliper. Tumor volume (mm^3^) was calculated as follows: volume = (shortest diameter)^2^ × (longest diameter) × 0.5. At the endpoint, tumors were harvested and weighed.

To investigate the synergistic effect of YTHDF1 depletion and chemoresistance, mice were treated with 5-fluorouracil (50 mg/kg, MCE #HY-90006) or oxaliplatin (7.5 mg/kg, MCE #HY-17371) intraperitoneally twice per week. VNP-siNC/*siYTHDF1* (0.5 OD per mouse injection intratumorally) was assembled as described and treatment was initiated when tumor volume reached an average of 100 mm^3^ at a frequency of every 2 days. SAC (Selleckchem, S0922) was administered intraperitoneally (50 mg/kg) twice per week when tumor volume reached an average of 100 mm^3^ at a frequency of every 2 days. All mice were sacrificed 4-6 weeks after injection. All animal experiments were approved by the Animal Experimentation Ethics Committee (Ref: 22-082-MIS) of The Chinese University of Hong Kong and Shenzhen Research Institute.

**RNA-sequencing**

Total RNAs from control or YTHDF1-overexpressed CSC28 were extracted with TRIzol (Invitrogen) and sent to Shanghai Jiayin Biotechnology Ltd, Shanghai, China for RNA sequencing. A total amount of 3 µg RNA per sample was used as input material for the RNA sample preparations. Sequencing libraries were generated using NEBNext® UltraTM RNA Library Prep Kit for Illumina® (NEB, USA) following the manufacturer’s recommendations, and index codes were added to attribute sequences to each sample. The clustering of the index-coded samples was performed on a cBot Cluster Generation System using TruSeq PE Cluster Kit v3-cBot-HS (Illumina) according to the manufacturer’s instructions. After cluster generation, the library preparations were sequenced on an Illumina Novaseq6000 platform and 150 bp paired-end reads were generated.

**Ribosome-sequencing**

To block translational elongation, cells with or without YTHDF1 overexpression were treated with 100 μg/ml cycloheximide (CHX) for 5 min at 37°C. The unprotected mRNA regions in the cells were excluded by treating with RNAse I. According to the ribosome-sequencing (Ribo-seq) protocol from the company (Gene Denovo, China), Ribo-seq libraries were constructed using NEBNext® Multiple Small RNA Library Prep Set for Illumina® (catalog no.E7300S, E7300L). The intact mRNA-ribosome complexes were sequenced by using Illumina HiSeq^TM^ X10. Reads mapping on human rRNAs, snoRNAs, snRNAs, and tRNAs from the GENCODE project (v30) were excluded. The residual reads were mapped on the human genome via bowtie2 (version 2.3.4.3) with option -L 10. The featureCounts (version 1.6.4) with the parameters (M –fracOverlap 0.4 –largestOverlap) was utilized for calculating the expression of protein-coding genes. Pathway analysis of Ribo-seq was applied via Gene Set Enrichment Analysis (GSEA) methods.

**MeRIP-sequencing**

Total RNAs from CSC28 were extracted with TRIzol (Invitrogen) and sent to Shanghai Jiayin Biotechnology Ltd, Shanghai, China for MeRIP-sequencing, mRNA was fragmented and then incubated with m6A antibody for immunoprecipitation. Immunoprecipitated RNA was analyzed through high-throughput sequencing. MeRIP-seq was performed as described previously.^1,2^ In brief, total RNA was isolated and fragmented into ~100-nucleotide-long fragments. Approximately 5% of fragmented RNA as input RNA, other RNA was analyzed by immunoprecipitation using affinity-purified anti-m6A polyclonal antibodies (ABE572, Millipore, Germany). Sequencing was carried out using an Illumina NovaSeq 6000 platform.

**MeRIP and RIP-qPCR**

MeRIP was performed using anti-m^6^A antibody (Merck Millipore, #ABE572), RIP was performed using an anti-YTHDF1 antibody (Proteintech #17479-1-AP) using EZMagna RIP™ RNA-Binding Protein Immunoprecipitation Kit (Sigma-Aldrich #17-701) according to the manufacturer’s instructions. Briefly, cell pellets were lysed with the RIP Lysis Buffer. The cell lysates were then incubated with magnetic beads bound with the anti-YTHDF1 antibody overnight at 4°C. The beads were then washed with the RIP Wash Buffer for a total of 6 times and the RNAs were released by digesting the antibody with proteinase K in 1% (w/v) SDS at 55°C for 30 min. The RNAs then were isolated with phenol: chloroform: isoamyl alcohol (Fisher #BP1754I-100) and precipitated by ethanol. The resuspended RNAs were then subjected to RT-qPCR as described above. The primer sequences are listed in **S Table 5**.

**Ribosome-nascent chain complex qPCR**

Ribosome nascent-chain complex-bound mRNA-qPCR (RNC-qPCR) was conducted as previously reported (43). Briefly, cells were pretreated with cycloheximide at the concentration of 100 μg/ml for 15 min at 37 °C, before being lysed in lysis buffer on ice for 30 min. Lysis buffer was prepared by adding 1% Triton X-100 into ribosome buffer (RB buffer) (20 mM HEPES-KOH [pH = 7.4], 15 mM MgCl2, 200 mM KCl, 100 mg/ml cycloheximide and 2 mM dithiothreitol). Cell lysate was extracted by centrifuging at 4 °C for 10 min at 16,200 g. 10% lysate was used as input control, another lysate was loaded onto 30% sucrose buffer and subsequently ultracentrifuged at 4 °C for 5 h at 174,900 g. The cell pellets were harvested and extracted by TRIzol. Total RNA isolated from the input control and RNC samples were used for RT-qPCR for further analysis.

**Cell Culture Media and Condition**

Cancer stem cell culture medium contained ﻿DMEM/F-12 (Thermo Fisher Scientific #11320033). supplemented with 1% antibiotic-antimycotic (Thermo Fisher Scientific #15240096), GlutaMAX (1:100, Thermo Fisher Scientific #35050061), HEPES (1:100, Thermo Fisher Scientific #15630080), MEM Non-Essential Amino Acids Solution (1:100, Thermo Fisher Scientific #11140050), Sodium Pyruvate (1:100, Thermo Fisher Scientific #11360070), N-2 Supplement (1:100, Thermo Fisher Scientific #17502048), NeuroCult™ SM1 Neuronal Supplement (1:250, STEMCELL Technologies #05711), Heparin (4 μg/ml, Sigma-Aldrich #H4784), Lipids (0.2%, Sigma-Aldrich #L0288), EGF (20 ng/ml, STEMCELL Technologies #78006.1) and basic FGF (10 ng/ml, STEMCELL Technologies #78003). Cancer stem cells are routinely sorted to enrich CD133^+^/LGR5^+^ populations using MojoSort™ Nanobeads. The choice of cell lines for *in vitro* and *in vivo* experiments was based on their differential YTHDF1 expression levels: LS174TS (low), CSC28 (moderate), and POP66 (high) **(Supplementary figure 5l)**.

Organoids were cultured in Corning Growth Factor Reduced Matrigel matrix (Corning) in advanced DMEM/F12 medium (Thermo Fisher Scientific #12634028) supplemented with 1% Penicillin-Streptomycin (Thermo Fisher Scientific #15140148), HEPES (10 µM; Thermo Fisher Scientific #15630106), GlutaMAX™ Supplement (1:100; Thermo Fisher Scientific #35050061), serum-free B-27™ Supplement (1: 50; Thermo Fisher Scientific #17504044), 1.25 mM N-Acetyl-L-cysteine (MilliporeSigma), 10 nM [Leu15]-Gastrin I human (MilliporeSigma), 50 ng/mL recombinant murine epithelial growth factor (Thermo Fisher Scientific), 100 ng/mL murine Noggin (Peprotech) and 0.5 µM A 83-01 (Tocris Bioscience). All cultures were maintained at 37°C in a humidified incubator with 5% CO2. Regular assessments were conducted to ensure they were free of mycoplasma contamination.

**Lentivirus Production**

HEK293T cells were used for lentivirus production. HEK293T cells in a 10-cm dish were transfected with 8 μg of plasmid DNA, 6 μg of psPAX2 (Addgene #12260), 2 μg of pMD2.G (Addgene #12259), and 36 μL of lipofectamine 2000 (Invitrogen #11668019). Media was changed 6 hours after transfection, and the supernatant was collected at 72 hours after transfection and filtered with 0.22 µm filter to remove cell debris. Two shRNAs targeting YTHDF1 (shYTHDF1-1: 5’-GATACAGTTCATGACAATGA-3’ and shYTHDF1-2: 5’-CAGGCTGGAGAATAACGACAA-3’) and a nontargeting RNA sequence (shCtrl: 5’-TTCTCCGAACGTGTCACGT-3’) serving as negative control were introduced using the pLKO.1-puro vector (Addgene #8453). Human-wide type YTHDF1 or YTHDF1 mutant (K395A, Y397A) was cloned into pLenti CMV Blast empty (Addgene #17486).

**siRNA transfection**

For siRNA transfection, we used Lipofectamine RNAiMAX reagent (Thermo Fisher #13778150) following the manufacturer’s protocol. Cells were transfected with siRNA for 72 h and harvested for qPCR and Western blot. siRNAs were purchased from GenePharma Co. Ltd (Shanghai, China). The sequencing of human YTHDF1 siRNA: sense (CCACUCAAACUCUUUCGGGTT), antisense (CCCGAAAGAGUUUGAGUGGAA); human NOTCH1 siRNA: sense (GGAUCCACUGUGAGAACAATT), antisense (UUGUUCUCACAGUGGAUCCTT).

**Reverse transcription-quantitative PCR (RT-qPCR)**

Total RNA was extracted from cells using TRIzol Reagent (ThermoFisher). RNA purity and concentration were measured by ND-1000 (NanoDrop Technologies). RNA (1 µg) was reverse transcribed using PrimeScript Reverse Transcription Master Mix (TaKaRa #RR036A). qPCR was performed with SYBR Green (Takara) in a QuantStudio Flex 7 Real-Time PCR system (Quantstudio Real-Time PCR software v1.7.2, Applied Biosystems) as follows: 10 min at 95 °C, and then 40 cycles of 15 s at 95 °C, 30 s at 60 °C and 30 s at 72 °C. All reactions were run in triplicates and normalized to GAPDH by the 2^−ΔΔCT^ method. Primer sequences are listed in **S Table 6**.

**MTT assay**

For cell viability assay, we used 3-(4,5-dimethylthiazol-2-yl)-2,5-diphenyltetrazolium (MTT, 5 mg/mL; Invitrogen). One thousand cells per well were seeded onto a 96-well plate. Cell viability was determined by incubation with MTT for 4 hours, followed by the addition of DMSO and measurement of absorbance at 570 nm. All experiments were conducted with 5 to 10 replicates per group.

**Apoptosis assays**

Apoptosis was evaluated by FITC Annexin V Apoptosis Detection Kit (BD Biosciences) according to manufacturer’s instructions. All stained cells were analyzed by flow cytometer (BD FACSCelesta Flow Cytometer, BD Biosciences). Data were analyzed by FlowJo (version 10.4; RRID: SCR_008520).

**Immunohistochemistry and Immunofluorescence**

Paraffin-embedded tissues were sectioned, deparaffinized with xylene, and rehydrated in alcohol. Antigen retrieval was performed in the microwave with sodium citrate buffer (10 mM sodium citrate, 0.05% tween 20, pH 6.0) for 30 min. Hydrogen peroxide (3%) was used to block the endogenous peroxidase activity. Slides were blocked with 5% bovine serum albumin (BSA) and incubated with primary antibodies in 1% BSA overnight. After washing, slides were incubated with rabbit on rodent HRP-polymer (BioCare Medical #RMR622L) for 40 min and then developed with DAB Plus (ThermoFisher). The sections were further counterstained with hematoxylin. Images were captured by light microscope equipped with Metafer Automatic Slide Scanning and Imaging System. The proportion of Ki-67-positive cells in each random field was measured by ImageJ with the plugin IHC Profiler.

For immunofluorescence, after antigen retrieval, the sections were further permeabilized with 0.25% Triton X-100 for 15 mins, blocked with 5% BSA, and incubated with primary antibodies in 1% BSA overnight at 4 ℃, followed by secondary incubation at room temperature for 1 hour. The sections were then washed with PBS twice, stained with Hoechst 33342 (Thermo Fisher Scientific #H3570), and further washed with PBS three times, followed by mounting with ProLong™ Glass Antifade Mountant (Thermo Fisher Scientific #P36980). The fluorescence images were acquired with the Leica TCS SP8 multiphoton system.

**Western blot**

Total proteins were extracted by CytoBuster Protein Extraction Reagent (Merck Millipore) containing protease and phosphatase inhibitors (Roche). Protein concentration was measured by Pierce BCA Protein Assay Kit (Thermo Fisher Scientific) according to the manufacturer’s instructions. 20-40 mg of the extracted proteins were separated by 10% SDS-PAGE gel electrophoresis (80-100V for 2 hours) and transferred onto PVDF membranes (100V for 2 hours) with pore size of 0.45 µm (Merck Millipore). Membranes were then blocked by 5% bovine serum albumin for 60 minutes, and incubated with primary antibodies at 4 ^°^C overnight, followed by 60 minutes of incubation of secondary antibodies at room temperature. Upon substantial wash by detergent (Tris-buffered saline with 0.1% Tween-20), proteins of interest were visualized by SuperSignal West Femto Maximum Sensitivity Substrate (Thermo Fisher Scientific) using ChemiDoc XRS+ System (Bio-Rad, Hercules, CA). Antibodies used for Western blot are listed in **S Table 2**.

**Luciferase reporter assay**

Cells were co-transfected with pmiRGLO-NOTCH1- 3′UTR or pmiRGLO-NOTCH1-Mut-3′ UTR in a 12-well plate, together with pRL-TK (RRID: Addgene_11313). At 24 hours post transfection, cells were lysed (Promega, E3971) and analyzed with the Dual-Glo Luciferase Assay (Promega). Luciferase (F-luc) activity was normalized to *Renilla*(R-luc) activity.

**Statistical analysis**

All measurements were performed in biological replicates. GraphPad Prism version 8 (GraphPad Software; San Diego, CA) was used for data analysis, and the data were shown as means ± s.d., unless stated otherwise. A *p*-value lower than 0.05 was regarded as statistically significant unless stated otherwise.

**References**

1. Chen, H., et al. RNA N^6^-methyladenosine methyltransferase METTL3 facilitates colorectal cancer by activating the m^6^A-GLUT1-mTORC1 axis and is a therapeutic target. *Gastroenterology* **160**, 1284-1300 (2021).

2. Zhai, J., et al. ALKBH5 drives immune suppression via targeting AXIN2 to promote colorectal cancer and is a target for boosting immunotherapy. *Gastroenterology* **165**, 445-462 (2023).


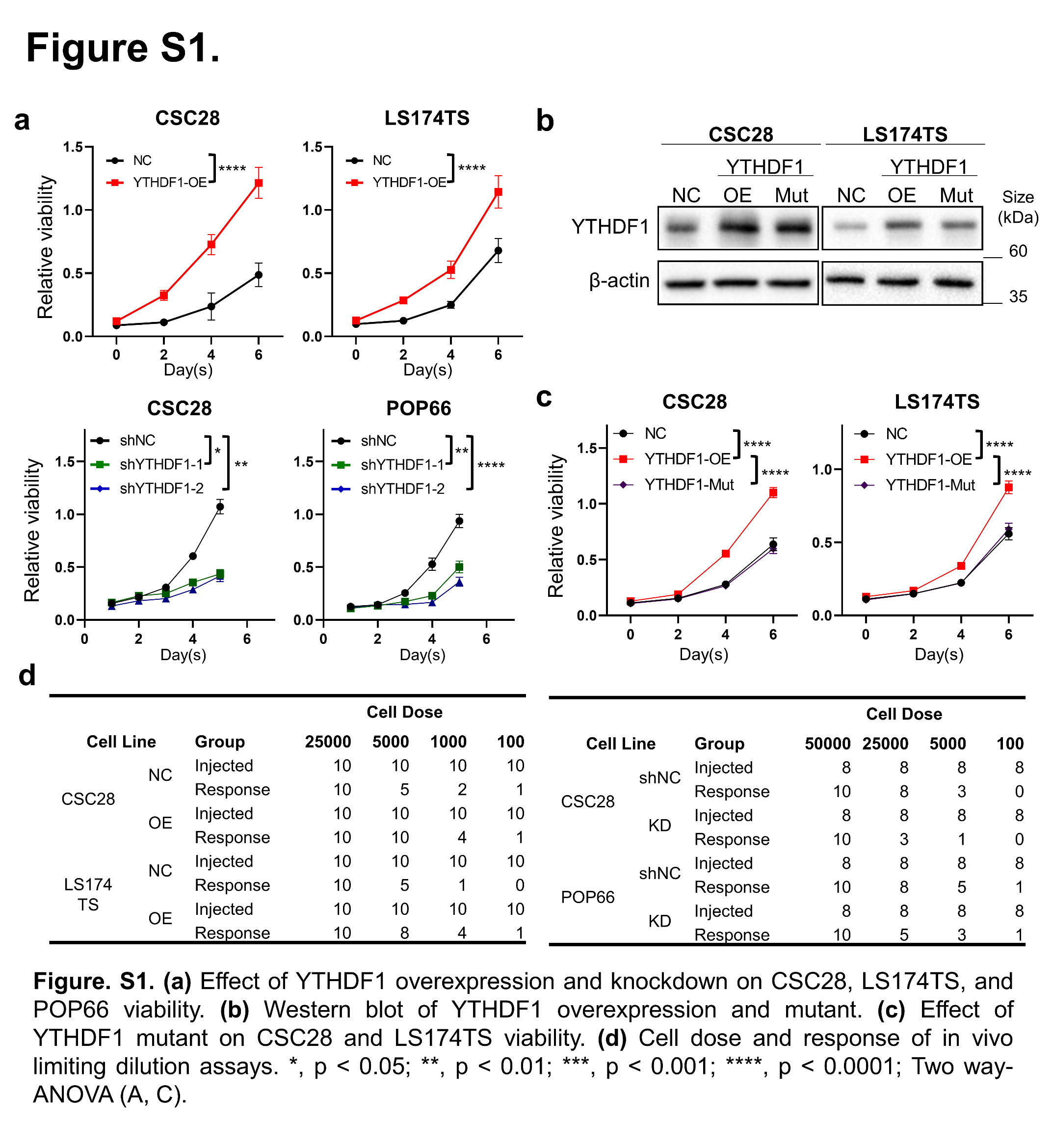


**Figure. S1. (a)** Effect of YTHDF1 overexpression and knockdown on CSC28, LS174TS, and POP66 viability. **(b)** Western blot of YTHDF1 overexpression and mutant. **(c)** Effect of YTHDF1 mutant on CSC28 and LS174TS viability. **(d)** Cell dose and response of in vivo limiting dilution assays. *, p < 0.05; **, p < 0.01; ***, p < 0.001; ****, p < 0.0001; Two way-ANOVA (A, C).


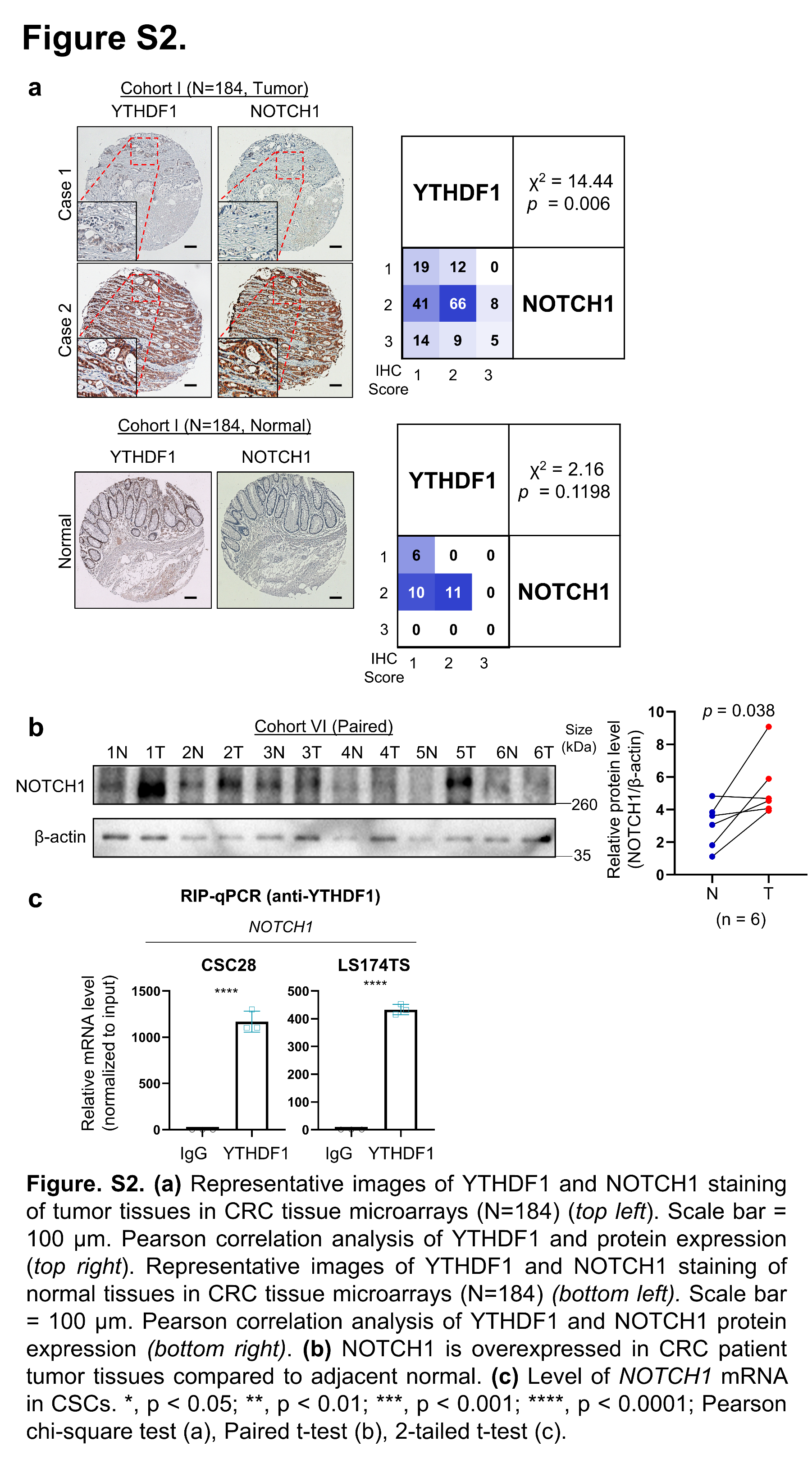


**Figure. S2. (a)** Representative images of YTHDF1 and NOTCH1 staining of tumor tissues in CRC tissue microarrays (N=184) (*top left*). Scale bar = 100 μm. Pearson correlation analysis of YTHDF1 and protein expression (*top right*). Representative images of YTHDF1 and NOTCH1 staining of normal tissues in CRC tissue microarrays (N=184) *(bottom left).* Scale bar = 100 μm. Pearson correlation analysis of YTHDF1 and NOTCH1 protein expression *(bottom right)*. **(b)** NOTCH1 is overexpressed in CRC patient tumor tissues compared to adjacent normal. **(c)** Level of *NOTCH1* mRNA in CSCs. *, p < 0.05; **, p < 0.01; ***, p < 0.001; ****, p < 0.0001; Pearson chi-square test (a), Paired t-test (b), 2-tailed t-test (c).


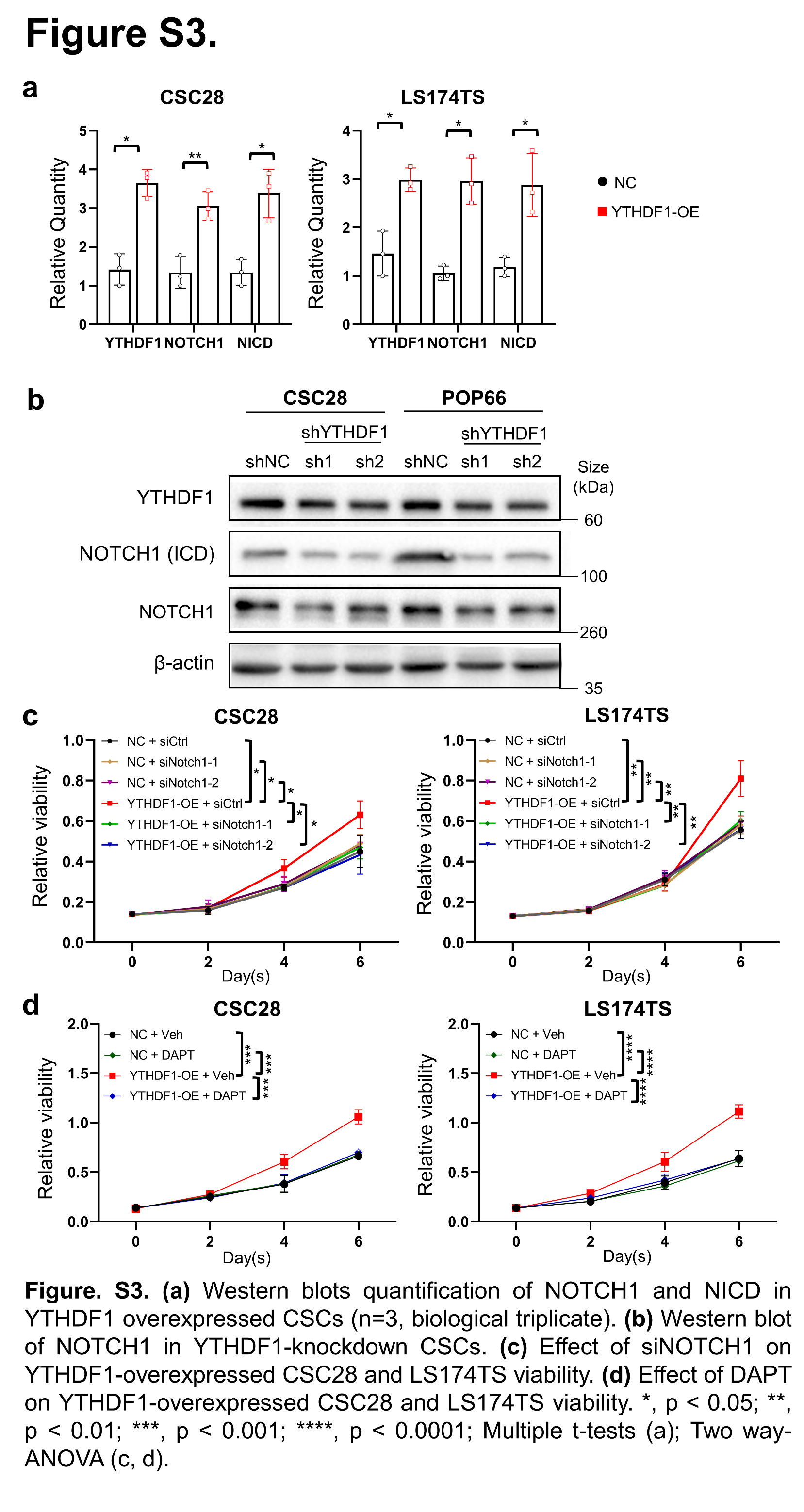


**Figure. S3. (a)** Western blots quantification of NOTCH1 and NICD in YTHDF1 overexpressed CSCs (n=3, biological triplicate). **(b)** Western blot of NOTCH1 in YTHDF1-knockdown CSCs. **(c)** Effect of siNOTCH1 on YTHDF1-overexpressed CSC28 and LS174TS viability. **(d)** Effect of DAPT on YTHDF1-overexpressed CSC28 and LS174TS viability. *, p < 0.05; **, p < 0.01; ***, p < 0.001; ****, p < 0.0001; Multiple t-tests (a); Two way-ANOVA (c, d).

**
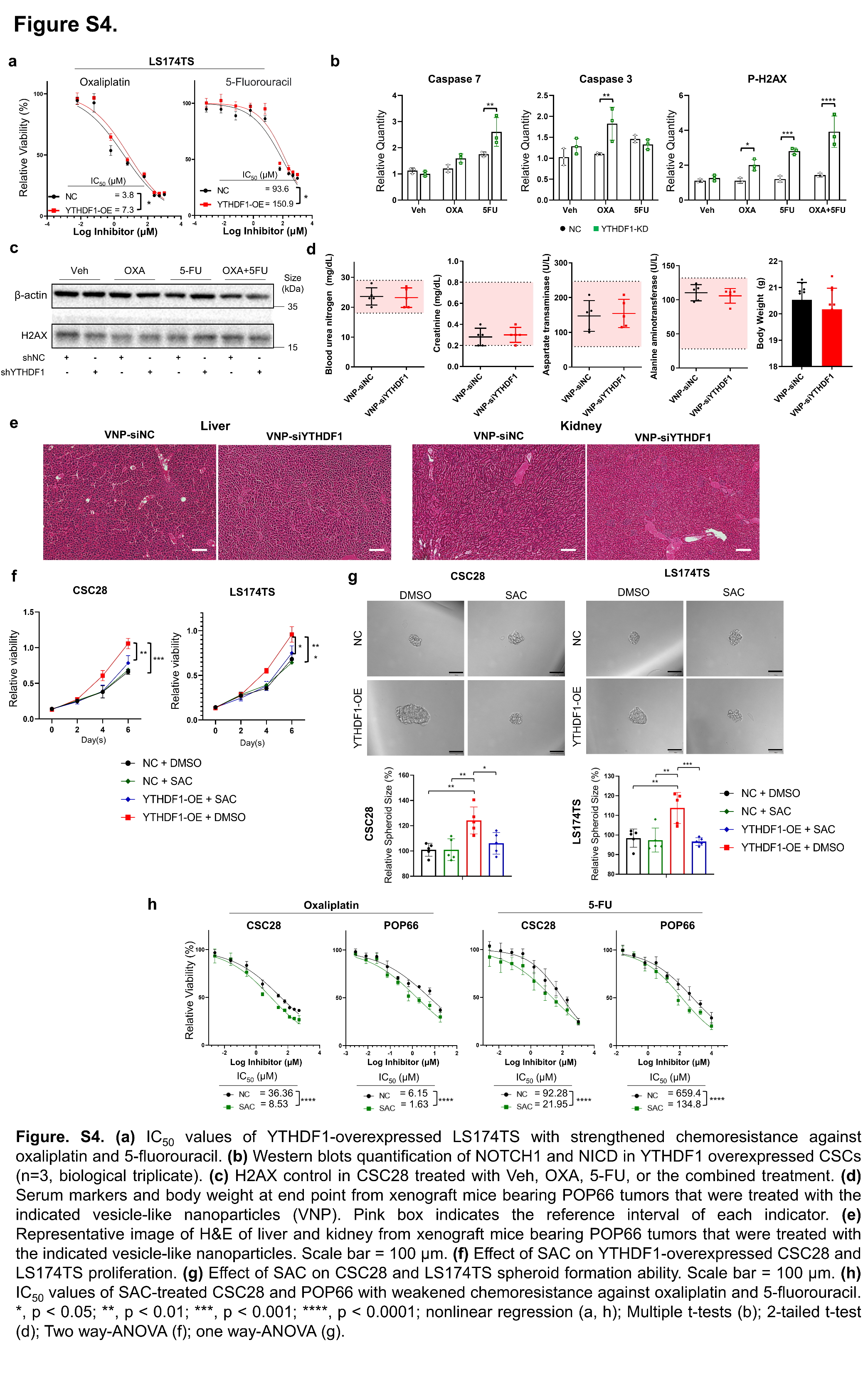
**

**Figure. S4. (a)** IC_50_ values of YTHDF1-overexpressed LS174TS with strengthened chemoresistance against oxaliplatin and 5-fluorouracil. **(b)** Western blots quantification of NOTCH1 and NICD in YTHDF1 overexpressed CSCs (n=3, biological triplicate). **(c)** H2AX control in CSC28 treated with Veh, OXA, 5-FU, or the combined treatment. **(d)** Serum markers and body weight at end point from xenograft mice bearing POP66 tumors that were treated with the indicated vesicle-like nanoparticles (VNP). Pink box indicates the reference interval of each indicator. **(e)** Representative image of H&E of liver and kidney from xenograft mice bearing POP66 tumors that were treated with the indicated vesicle-like nanoparticles. Scale bar = 100 μm. **(f)** Effect of SAC on YTHDF1-overexpressed CSC28 and LS174TS proliferation. **(g)** Effect of SAC on CSC28 and LS174TS spheroid formation ability. Scale bar = 100 μm. **(h)** IC_50_ values of SAC-treated CSC28 and POP66 with weakened chemoresistance against oxaliplatin and 5-fluorouracil. *, p < 0.05; **, p < 0.01; ***, p < 0.001; ****, p < 0.0001; nonlinear regression (a, h); Multiple t-tests (b); 2-tailed t-test (d); Two way-ANOVA (f); one way-ANOVA (g).


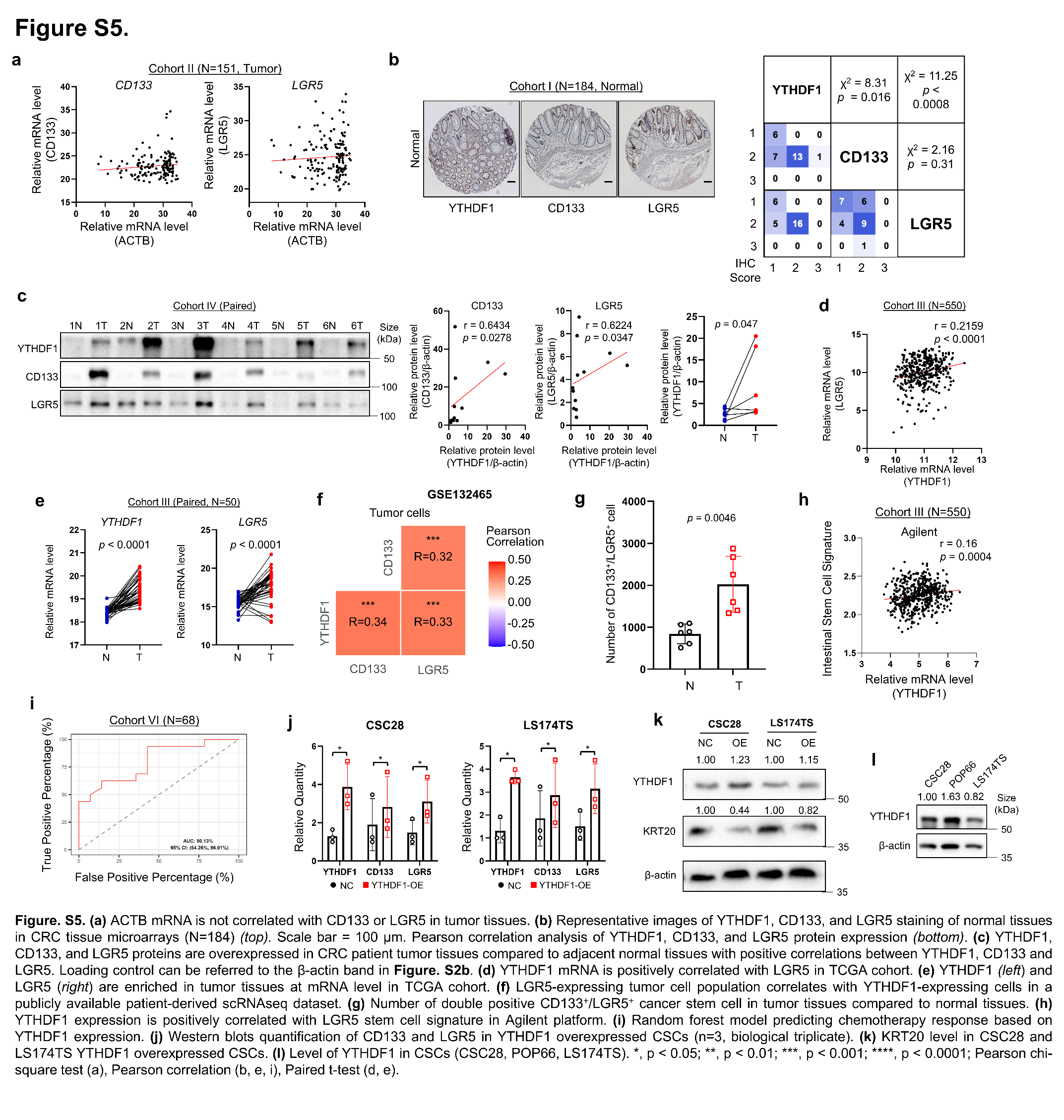


**Figure. S5. (a)** ACTB mRNA is not correlated with CD133 or LGR5 in tumor tissues. **(b)** Representative images of YTHDF1, CD133, and LGR5 staining of normal tissues in CRC tissue microarrays (N=184) *(top).* Scale bar = 100 μm. Pearson correlation analysis of YTHDF1, CD133, and LGR5 protein expression *(bottom)*. **(c)** YTHDF1, CD133, and LGR5 proteins are overexpressed in CRC patient tumor tissues compared to adjacent normal tissues with positive correlations between YTHDF1, CD133 and LGR5. **(d)** YTHDF1 mRNA is positively correlated with LGR5 in TCGA cohort. **(e)** YTHDF1 *(left*) and LGR5 (*right*) are enriched in tumor tissues at mRNA level in TCGA cohort. **(f)** LGR5-expressing tumor cell population correlates with YTHDF1-expressing cells in a publicly available patient-derived scRNAseq dataset. **(g)** Number of double positive CD133^+^/LGR5^+^ cancer stem cell in tumor tissues compared to normal tissues. **(h)** YTHDF1 expression is positively correlated with LGR5 stem cell signature in Agilent platform. **(i)** Random forest model predicting chemotherapy response based on YTHDF1 expression. **(j)** Western blots quantification of CD133 and LGR5 in YTHDF1 overexpressed CSCs (n=3, biological triplicate). **(k)** KRT20 level in CSC28 and LS174TS YTHDF1 overexpressed CSCs. **(l)** Level of YTHDF1 in CSCs (CSC28, POP66, LS174TS). *, p < 0.05; **, p < 0.01; ***, p < 0.001; ****, p < 0.0001; Pearson chi-square test (a), Pearson correlation (b, e, i), Paired t-test (d, e).

**Table S1. Clinical features YTHDF1 in CRC patients (Cohort I)**

| Clinical feature | High expression (n=104) | % | Low expression (n=80) | % |
| --- | --- | --- | --- | --- |
| Age | 57.9±11.8 |  | 59.7±12.4 |  |
| Gender |  |  |  |  |
| M | 61 | 58.7 | 42 | 52.5 |
| F | 43 | 41.3 | 38 | 47.5 |
| TNM |  |  |  |  |
| I | 5 | 4.8 | 11 | 13.7 |
| II | 38 | 36.5 | 34 | 42.5 |
| III | 57 | 54.8 | 30 | 37.5 |
| IV | 4 | 3.9 | 5 | 6.3 |
| Localization |  |  |  |  |
| Colon | 57 | 54.8 | 42 | 52.5 |
| Rectum | 47 | 45.2 | 38 | 47.5 |
| Differentiation |  |  |  |  |
| Low | 31 | 29.8 | 22 | 27.5 |
| High | 73 | 70.2 | 58 | 72.5 |

**Table S2. Clinical features of chemotherapy Responders and Non-Responders in CRC patients (Cohort V)**

This cohort contains 83 patients with unresectable CRC treated with FOLFOX therapy between April 2007 and December 2010 at Teikyo University Hospital at Mizonokuchi and Gifu University Hospital. CRC samples, collected prior to mFOLFOX6 therapy, included 56 primary tumors and 27 metastatic lesions (23 liver, 1 lung, and 3 peritoneal). None of the patients had received prior chemotherapy or radiotherapy. The mFOLFOX6 regimen consisted of 85 mg/m² oxaliplatin, 200 mg/m² leucovorin, and 400 mg/m² 5-FU bolus on day 1, followed by 2400 mg/m² 5-FU as a 46-hour continuous infusion, repeated every 2 weeks. After four cycles, tumor response was evaluated by computed tomography and classified according to modified Response Evaluation Criteria in Solid Tumors (RECIST) as complete response (CR, complete disappearance of all target lesions), partial response (PR, ≥10% decrease in the sum of the longest diameters of target lesions), progressive disease (PD, ≥10% increase in the sum of the longest diameters of target lesions), or stable disease (SD, neither sufficient shrinkage for PR nor increase for PD). (M, male; F, female; CR, complete response; PR, partial response; SD, stable disease; PD, progressive disease.)

| Accession | Gender | Tumor location | Lesion | Regimen | Response status | Response category |
| --- | --- | --- | --- | --- | --- | --- |
| GSM710801 | F | Liver | Metastasis | mFOLFOX6 | Responder | PR |
| GSM710802 | M | Rectum | Primary | mFOLFOX6 | Responder | PR |
| GSM710803 | M | Descending | Primary | mFOLFOX6 | Responder | PR |
| GSM710804 | M | Transverse | Primary | mFOLFOX6 | Responder | PR |
| GSM710805 | M | Rectum | Primary | mFOLFOX6 | Responder | PR |
| GSM710806 | F | Cecum | Primary | mFOLFOX6 | Responder | PR |
| GSM710807 | M | Ascending | Primary | mFOLFOX6 | Responder | PR |
| GSM710808 | M | Rectum | Primary | mFOLFOX6 | Responder | PR |
| GSM710809 | M | Rectum | Primary | mFOLFOX6 | Responder | PR |
| GSM710810 | M | Sigmoid | Primary | mFOLFOX6 | Responder | PR |
| GSM710811 | M | Sigmoid | Primary | mFOLFOX6 | Responder | PR |
| GSM710812 | M | Rectum | Primary | mFOLFOX6 | Responder | PR |
| GSM710813 | M | Peritoneum | Metastasis | mFOLFOX6 | Responder | PR |
| GSM710814 | M | Liver | Metastasis | mFOLFOX6 | Responder | PR |
| GSM710815 | M | Liver | Metastasis | mFOLFOX6 | Responder | PR |
| GSM710816 | M | Peritoneum | Metastasis | mFOLFOX6 | Responder | CR |
| GSM710817 | M | Peritoneum | Metastasis | mFOLFOX6 | Responder | PR |
| GSM710818 | F | Liver | Metastasis | mFOLFOX6 | Responder | PR |
| GSM710819 | M | Liver | Metastasis | mFOLFOX6 | Responder | PR |
| GSM710820 | F | Liver | Metastasis | mFOLFOX6 | Responder | CR |
| GSM710821 | M | Rectum | Primary | mFOLFOX6 | Responder | PR |
| GSM710822 | M | Ascending | Primary | mFOLFOX6 | Responder | PR |
| GSM710823 | M | Ascending | Primary | mFOLFOX6 | Responder | PR |
| GSM710824 | M | Sigmoid | Primary | mFOLFOX6 | Responder | PR |
| GSM710825 | F | Rectum | Primary | mFOLFOX6 | Responder | PR |
| GSM710826 | F | Rectum | Primary | mFOLFOX6 | Responder | PR |
| GSM710827 | F | Ascending | Primary | mFOLFOX6 | Responder | PR |
| GSM710828 | F | Liver | Metastasis | mFOLFOX6 | Non-Responder | PD |
| GSM710829 | M | Liver | Metastasis | mFOLFOX6 | Non-Responder | PD |
| GSM710830 | M | Rectum | Primary | mFOLFOX6 | Non-Responder | PD |
| GSM710831 | M | Cecum | Primary | mFOLFOX6 | Non-Responder | SD |
| GSM710832 | M | Ascending | Primary | mFOLFOX6 | Non-Responder | SD |
| GSM710833 | M | Ascending | Primary | mFOLFOX6 | Non-Responder | PD |
| GSM710834 | M | Ascending | Primary | mFOLFOX6 | Non-Responder | SD |
| GSM710835 | M | Rectum | Primary | mFOLFOX6 | Non-Responder | SD |
| GSM710836 | F | Rectum | Primary | mFOLFOX6 | Non-Responder | SD |
| GSM710837 | M | Rectum | Primary | mFOLFOX6 | Non-Responder | SD |
| GSM710839 | M | Liver | Metastasis | mFOLFOX6 | Non-Responder | PD |
| GSM710841 | M | Liver | Metastasis | mFOLFOX6 | Non-Responder | SD |
| GSM710843 | M | Liver | Metastasis | mFOLFOX6 | Non-Responder | SD |
| GSM710845 | M | Lung | Metastasis | mFOLFOX6 | Non-Responder | SD |
| GSM710846 | M | Liver | Metastasis | mFOLFOX6 | Non-Responder | PD |
| GSM710849 | M | Liver | Metastasis | mFOLFOX6 | Non-Responder | PD |
| GSM710853 | F | Liver | Metastasis | mFOLFOX6 | Non-Responder | SD |
| GSM710855 | F | Liver | Metastasis | mFOLFOX6 | Non-Responder | SD |
| GSM710858 | M | Liver | Metastasis | mFOLFOX6 | Non-Responder | PD |
| GSM710860 | M | Liver | Metastasis | mFOLFOX6 | Non-Responder | PD |
| GSM710862 | F | Sigmoid | Primary | mFOLFOX6 | Non-Responder | SD |
| GSM710863 | M | Rectum | Primary | mFOLFOX6 | Non-Responder | SD |
| GSM710865 | F | Sigmoid | Primary | mFOLFOX6 | Non-Responder | PD |
| GSM710867 | M | Sigmoid | Primary | mFOLFOX6 | Non-Responder | SD |
| GSM710869 | F | Sigmoid | Primary | mFOLFOX6 | Non-Responder | SD |
| GSM710871 | M | Rectum | Primary | mFOLFOX6 | Non-Responder | PD |
| GSM710873 | M | Rectum | Primary | mFOLFOX6 | Non-Responder | PD |
| GSM710875 | F | Rectum | Primary | mFOLFOX6 | Responder | PR |
| GSM710877 | M | Sigmoid | Primary | mFOLFOX6 | Responder | PR |
| GSM710879 | F | Sigmoid | Primary | mFOLFOX6 | Responder | PR |
| GSM710881 | F | Liver | Metastasis | mFOLFOX6 | Responder | PR |
| GSM710883 | M | Liver | Metastasis | mFOLFOX6 | Responder | PR |
| GSM710885 | M | Sigmoid | Primary | mFOLFOX6 | Responder | PR |
| GSM710886 | M | Descending | Primary | mFOLFOX6 | Responder | PR |
| GSM710888 | M | Rectum | Primary | mFOLFOX6 | Responder | PR |
| GSM710890 | M | Rectum | Primary | mFOLFOX6 | Responder | PR |
| GSM710892 | F | Rectum | Primary | mFOLFOX6 | Responder | PR |
| GSM710894 | F | Sigmoid | Primary | mFOLFOX6 | Responder | PR |
| GSM710896 | F | Rectum | Primary | mFOLFOX6 | Responder | PR |
| GSM710898 | F | Sigmoid | Primary | mFOLFOX6 | Responder | PR |
| GSM710900 | M | Cecum | Primary | mFOLFOX6 | Responder | PR |
| GSM710902 | F | Ascending | Primary | mFOLFOX6 | Responder | PR |
| GSM710905 | F | Sigmoid | Primary | mFOLFOX6 | Non-Responder | PD |
| GSM710906 | M | Sigmoid | Primary | mFOLFOX6 | Non-Responder | SD |
| GSM710908 | F | Rectum | Primary | mFOLFOX6 | Non-Responder | SD |
| GSM710911 | M | Rectum | Primary | mFOLFOX6 | Non-Responder | SD |
| GSM710913 | M | Liver | Metastasis | mFOLFOX6 | Non-Responder | PD |
| GSM710915 | M | Liver | Metastasis | mFOLFOX6 | Non-Responder | PD |
| GSM710916 | F | Liver | Metastasis | mFOLFOX6 | Non-Responder | PD |
| GSM710918 | F | Liver | Metastasis | mFOLFOX6 | Non-Responder | PD |
| GSM710920 | F | Rectum | Primary | mFOLFOX6 | Non-Responder | PD |
| GSM710922 | M | Sigmoid | Primary | mFOLFOX6 | Non-Responder | SD |
| GSM710924 | F | Ascending | Primary | mFOLFOX6 | Non-Responder | PD |
| GSM710926 | F | Cecum | Primary | mFOLFOX6 | Non-Responder | PD |
| GSM710928 | M | Sigmoid | Primary | mFOLFOX6 | Non-Responder | SD |
| GSM710930 | M | Ascending | Primary | mFOLFOX6 | Non-Responder | PD |

**Table S3. Clinical features of chemotherapy Responders and Non-Responders in CRC patients (Cohort VI)**

Tumor samples were obtained from 68 untreated primary CRC tumors in patients with synchronous or metachronous liver metastases. Inclusion criteria included: histologically confirmed colon adenocarcinoma, advanced and bidimensionally measurable disease, age 18–75 years, and WHO performance status of 2 or lower. All patients underwent primary tumor resection or endoscopic biopsy prior to chemotherapy. Patients received various first-line treatment regimens, and tumor response was assessed using RECIST 1.0 criteria. The best observed response to first-line treatment was used to categorize patients (M, male; F, female; CR, complete response; PR, partial response; SD, stable disease; PD, progressive disease).

| Accession | Gender | Tumor location | Synchronous metastasis | Regimen | Response status | Response category |
| --- | --- | --- | --- | --- | --- | --- |
| GSM1875897 | M | Left colon | Yes | FOLFOX | Responder | PR |
| GSM1875898 | M | Left colon | Yes | FOLFOX | Responder | PR |
| GSM1875899 | F | Right colon | Yes | FOLFOX | Non-Responder | PD |
| GSM1875900 | F | Right colon | Yes | FOLFOX | Responder | PR |
| GSM1875901 | F | Left colon | Yes | FOLFIRI | Responder | PR |
| GSM1875902 | F | Rectum-sigmoid junction | Yes | FOLFOX | Responder | PR |
| GSM1875903 | M | Caecum | Yes | FOLFIRI | Non-Responder | SD |
| GSM1875904 | M | Left colon | No | FOLFIRI | Responder | CR |
| GSM1875905 | F | Right colon | Yes | FOLFIRI | Non-Responder | SD |
| GSM1875906 | M | Left colon | Yes | FOLFIRI | Non-Responder | SD |
| GSM1875907 | M | Rectum | Yes | FOLFOX | Non-Responder | SD |
| GSM1875908 | M | Left colon | Yes | FOLFIRI | Non-Responder | SD |
| GSM1875909 | M | Left colon | Yes | FOLFIRI | Responder | PR |
| GSM1875910 | M | Transverse colon | Yes | FOLFIRI | Non-Responder | SD |
| GSM1875911 | F | Left colon | Yes | FOLFIRI | Responder | CR |
| GSM1875912 | F | Rectum | Yes | FOLFIRI | Responder | PR |
| GSM1875913 | F | Rectum | Yes | FOLFIRI | Non-Responder | SD |
| GSM1875914 | M | Rectum | Yes | FOLFOX | Responder | PR |
| GSM1875915 | M | Rectum-sigmoid junction | Yes | FOLFIRI | Responder | CR |
| GSM1875916 | F | Left colon | Yes | FOLFOX | Responder | PR |
| GSM1875917 | M | Rectum | Yes | FOLFOX | Non-Responder | SD |
| GSM1875918 | M | Left colon | Yes | FOLFOX | Responder | PR |
| GSM1875919 | M | Left colon | Yes | FOLFOX | Responder | PR |
| GSM1875920 | M | Rectum-sigmoid junction | Yes | FOLFOX | Responder | PR |
| GSM1875921 | M | Rectum | Yes | FOLFIRI | Non-Responder | PD |
| GSM1875922 | M | Left colon | Yes | FOLFIRI | Non-Responder | SD |
| GSM1875923 | M | Left colon | Yes | FOLFOX | Responder | PR |
| GSM1875924 | F | Rectum | Yes | FOLFOX | Responder | PR |
| GSM1875925 | F | Right colon | Yes | FOLFIRI | Non-Responder | PD |
| GSM1875926 | F | Rectum-sigmoid junction | Yes | FOLFIRI | Non-Responder | SD |
| GSM1875927 | M | Left colon | Yes | FOLFIRI | Non-Responder | SD |
| GSM1875928 | M | Rectum | Yes | FOLFIRI | Non-Responder | SD |
| GSM1875929 | F | Left colon | Yes | FOLFOX | Responder | PR |
| GSM1875930 | F | Rectum-sigmoid junction | Yes | FOLFIRI | Non-Responder | PD |
| GSM1875931 | M | Rectum-sigmoid junction | Yes | FOLFIRI | Responder | PR |
| GSM1875932 | F | Left colon | Yes | FOLFOX | Responder | PR |
| GSM1875933 | F | Left colon | Yes | FOLFIRI | Responder | PR |
| GSM1875934 | M | Left colon | Yes | FOLFIRI | Non-Responder | SD |
| GSM1875935 | M | Rectum | Yes | FOLFOX | Non-Responder | SD |
| GSM1875936 | F | Right colon | Yes | FOLFIRI | Responder | PR |
| GSM1875937 | M | Left colon | Yes | FOLFOX | Non-Responder | SD |
| GSM1875938 | F | Left colon | Yes | FOLFOX | Non-Responder | PD |
| GSM1875939 | M | Left colon | Yes | FOLFIRI | Non-Responder | SD |
| GSM1875940 | M | Left colon | Yes | FOLFIRI | Responder | PR |
| GSM1875941 | M | Left colon | Yes | FOLFIRI | Responder | PR |
| GSM1875942 | M | Right colon | Yes | FOLFIRI | Responder | PR |
| GSM1875943 | M | Left colon | Yes | FOLFIRI | Responder | PR |
| GSM1875944 | M | Right colon | Yes | FOLFIRI | Non-Responder | PD |
| GSM1875945 | F | Caecum | No | FOLFIRI | Responder | PR |
| GSM1875946 | M | Left colon | Yes | FOLFIRI | Responder | PR |
| GSM1875947 | F | Right colon | Yes | FOLFOX | Non-Responder | PD |
| GSM1875948 | M | Caecum | Yes | FOLFOX | Responder | CR |
| GSM1875949 | F | Left colon | Yes | FOLFIRI | Non-Responder | SD |
| GSM1875950 | M | Left colon | Yes | FOLFIRI | Responder | PR |
| GSM1875951 | M | Rectum | Yes | FOLFIRI | Non-Responder | SD |
| GSM1875952 | M | Left colon | Yes | FOLFOX | Non-Responder | SD |
| GSM1875953 | F | Right colon | Yes | FOLFIRI | Non-Responder | PD |
| GSM1875954 | F | Left colon | Yes | FOLFOX | Responder | PR |
| GSM1875955 | M | Left colon | No | FOLFOX | Responder | PR |
| GSM1875956 | F | Right colon | Yes | FOLFOX | Responder | PR |
| GSM1875957 | M | Right colon | Yes | FOLFIRI | Responder | CR |
| GSM1875958 | M | Left colon | No | FOLFIRI | Responder | CR |
| GSM1875959 | F | Right colon | No | FOLFOX | Non-Responder | PD |
| GSM1875960 | M | Rectum-sigmoid junction | No | FOLFIRI | Non-Responder | PD |
| GSM1875961 | M | Left colon | Yes | FOLFIRI | Non-Responder | SD |
| GSM1875962 | M | Rectum | Yes | FOLFIRI | Responder | CR |
| GSM1875963 | M | Right colon | Yes | FOLFIRI | Responder | PR |
| GSM1875964 | F | Left colon | Yes | FOLFIRI | Non-Responder | PD |

**Table S4. List of Antibodies**

| **Immunohistochemistry** | | |
| --- | --- | --- |
| Primary Antibody | Dilution | Catalog No. |
| YTHDF1 | 1:250 | Proteintech #17479-1-AP |
| LGR5 | 1:200 | Origene #TA503316 |
| CD133 | 1:200 | CST #64326 |
| NOTCH1 | 1:150 | CST #3608 |

| **Western blot** | | |
| --- | --- | --- |
| Primary Antibody | Dilution | Catalog No. |
| YTHDF1 | 1:1000 | Proteintech #17479-1-AP |
| LGR5 | 1:1000 | Origene #TA503316 |
| CD133 | 1:1000 | CST #64326 |
| NOTCH1 | 1:1000 | CST #3608 |
| Phospho-H2AX | 1:1000 | CST #2577 |
| Caspase 3 | 1:1000 | CST #9662 |
| Caspase 7 | 1:1000 | CST #9492 |
| β-actin | 1:1000 | CST #4970 |

**Table S5. Primer sequences for MeRIP and RIP-qPCR**

| **Primer name** | **Forward/Reverse** | **Sequence (5’ to 3’)** |
| --- | --- | --- |
| NOTCH1 - 1 | Forward | GGACGTCAGACTTGGCTCAG |
| NOTCH1 - 1 | Reverse | ACATCTTGGGACGCATCTGG |
| NOTCH1 - 2 | Forward | CCACCCCTCCTAGTTTGGGA |
| NOTCH1 - 2 | Reverse | TGGCATGACACACAACAGAC |
| NOTCH1 - 3 | Forward | CCAGTGAGGGACGTCAGACT |
| NOTCH1 - 3 | Reverse | TCAACATCTTGGGACGCATCT |

**Table S6. Primer sequences for qPCR**

| **Species** | **Target gene** | **Forward/Reverse** | **Sequence (5’ to 3’)** |
| --- | --- | --- | --- |
| Homo sapiens | ALDH1A1 | Forward | CAACAGAGGTTGGCAAGTTG |
| Homo sapiens | ALDH1A1 | Reverse | ACCCCATGGTGTGCAAATTC |
| Homo sapiens | CD133 | Forward | CCATAAAGCTGGACCCATTG |
| Homo sapiens | CD133 | Reverse | TTTTGGATTCATATGCCTTC |
| Homo sapiens | CD44 | Forward | AGCACAATCCAGGCAACTCC |
| Homo sapiens | CD44 | Reverse | CTGGTATGAGCTGAGGCTGC |
| Homo sapiens | EPHB2 | Forward | AGCGGCAAGATGTACTTCCA |
| Homo sapiens | EPHB2 | Reverse | ACACGATGGCGATGACAACC |
| Homo sapiens | EPHB3 | Forward | GAGTTGGCGTGGACATCTCA |
| Homo sapiens | EPHB3 | Reverse | GACTCGCGCACATTACACAC |
| Homo sapiens | GAPDH | Forward | AGAAGGCTGGGGCTCATTTG |
| Homo sapiens | GAPDH | Reverse | GAGGGGCCATCCACAGTCTT |
| Homo sapiens | LGR5 | Forward | TCCGCTTCCTGGAGGAGTTA |
| Homo sapiens | LGR5 | Reverse | CATCCAGACGCAGGGATTGA |
| Homo sapiens | Notch1 | Forward | GGACGTCAGACTTGGCTCAG |
| Homo sapiens | Notch1 | Reverse | ACATCTTGGGACGCATCTGG |
| Homo sapiens | Sox9 | Forward | GAGGAAGTCGGTGAAGAAC |
| Homo sapiens | Sox9 | Reverse | CCTCTCGCTTCAGGTCAGC |
| Homo sapiens | YTHDF1 | Forward | CAAGCACACAACCTCCATCT |
| Homo sapiens | YTHDF1 | Reverse | GTAAGAAACTGGTTCGCCCT |
