# Supplementary figures and images for: YTHDF1 targets the chemotherapy response by suppressing NOTCH1-induced stemness in colorectal cancer
Source: Signal Transduct Target Ther. 2025 Dec 22;10:409. doi: 10.1038/s41392-025-02507-1 (PMC12719390; doi:10.1038/s41392-025-02507-1)

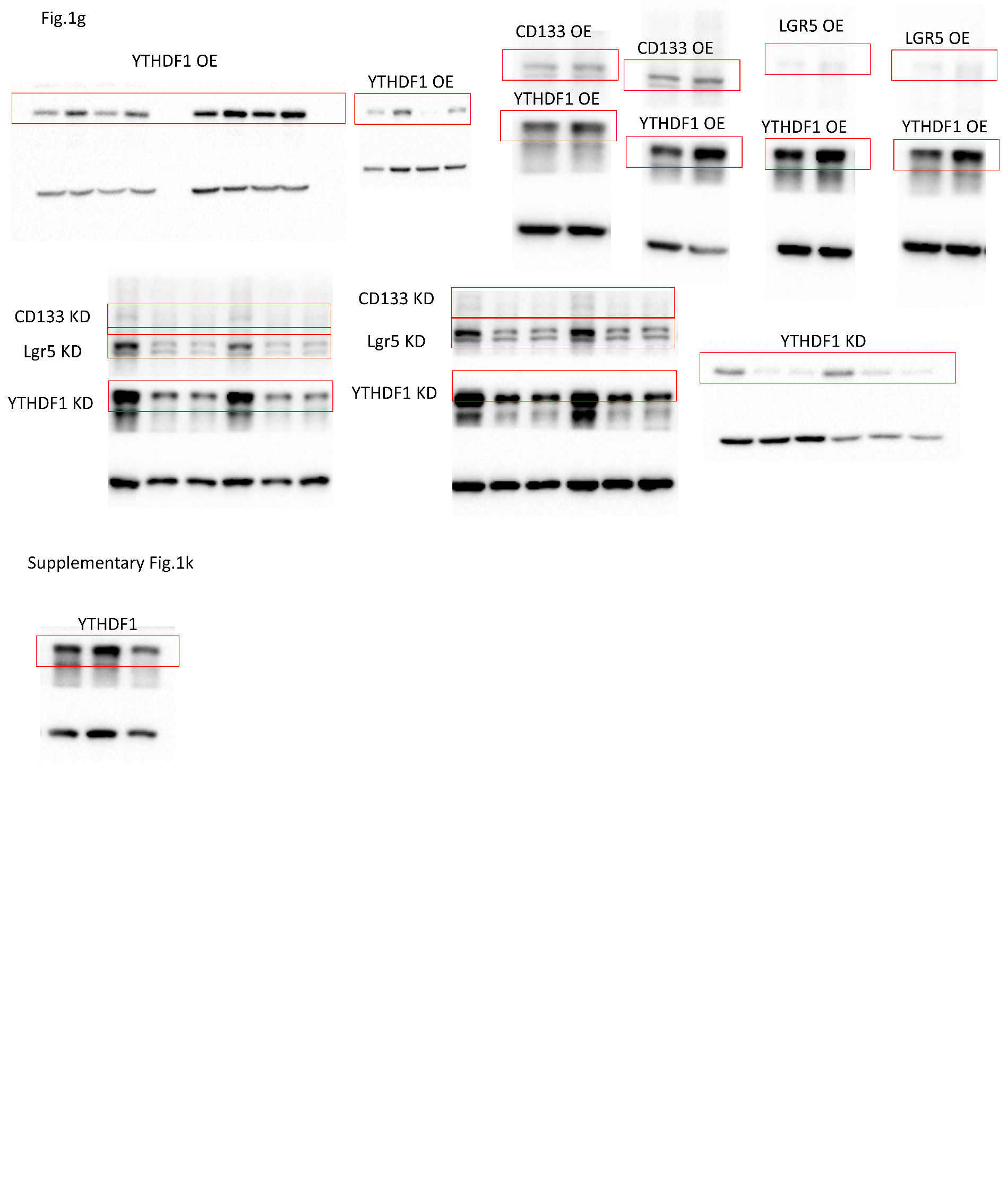


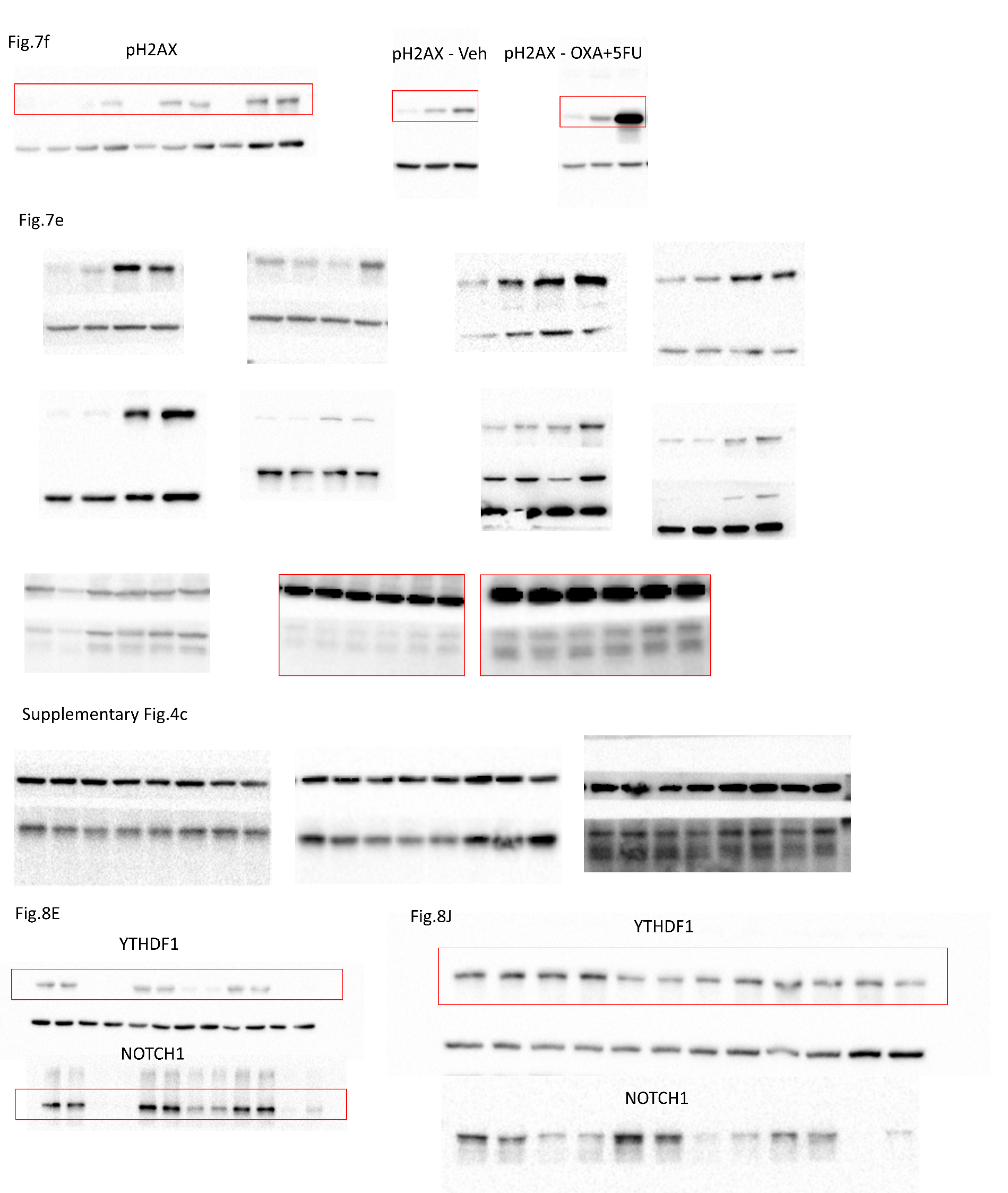

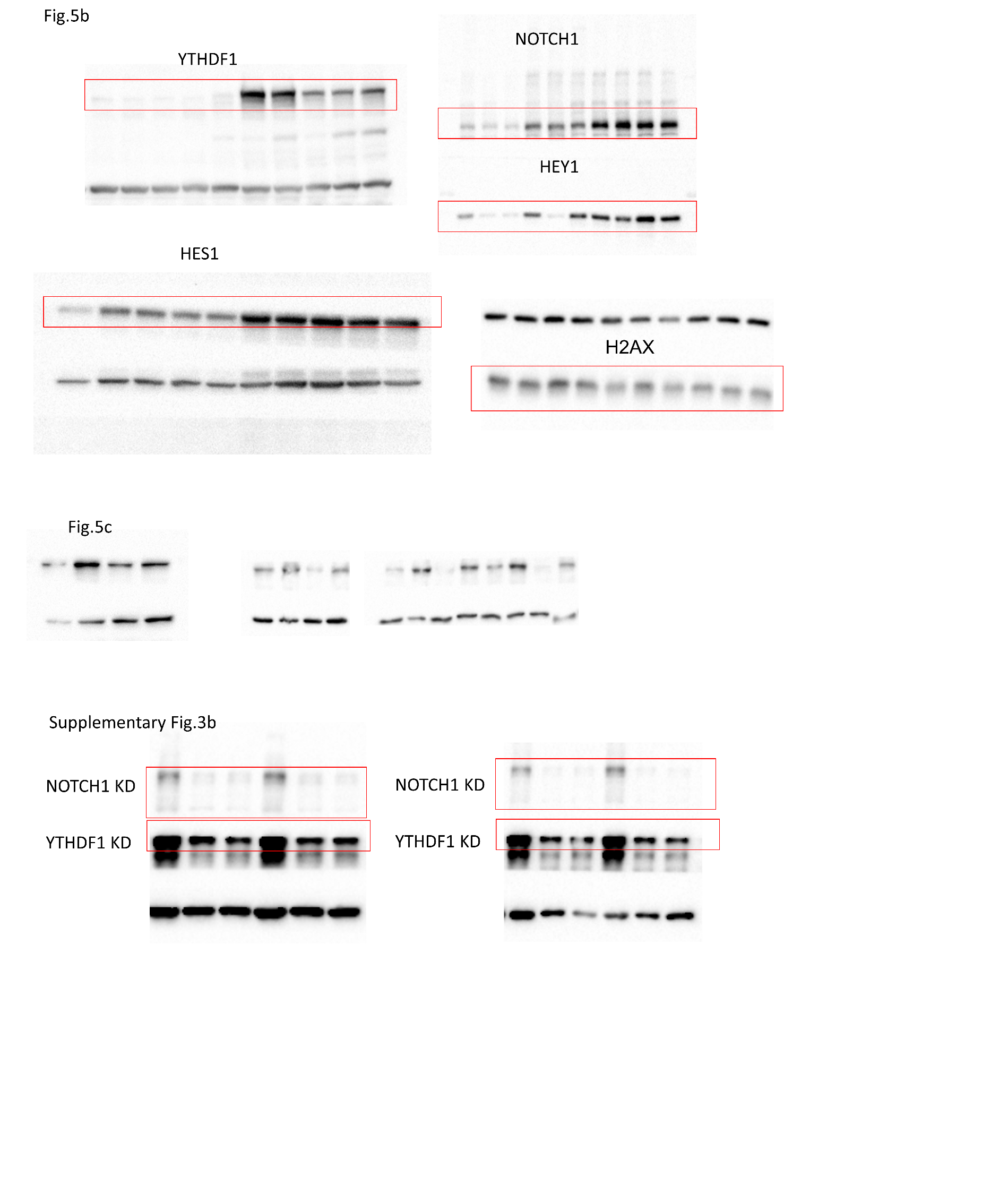

Supplement: Supplementary file 2 — Western blot raw data [file 41392_2025_2507_MOESM2_ESM.docx]
