# Supplementary material for: YTHDF1 targets the chemotherapy response by suppressing NOTCH1-induced stemness in colorectal cancer
Source: Signal Transduct Target Ther. 2025 Dec 22;10:409. doi: 10.1038/s41392-025-02507-1 (PMC12719390; doi:10.1038/s41392-025-02507-1)
Supplement: Supplementary file 3 — Raw data for rebuttal [file 41392_2025_2507_MOESM3_ESM.pdf]

Fig. 4f (1.0 and 1.1)

Original image

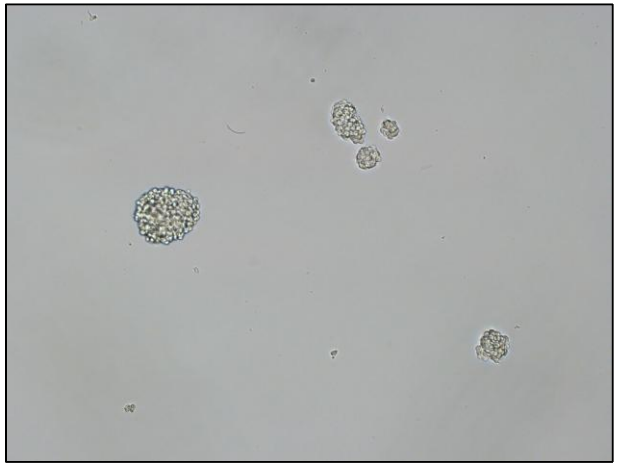

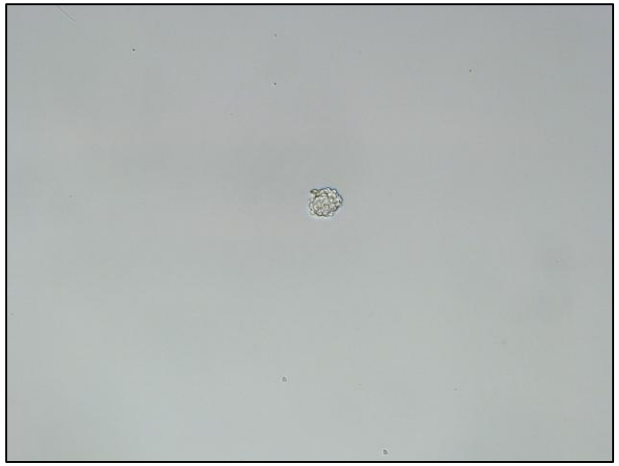

Fig. 5f (2.0)

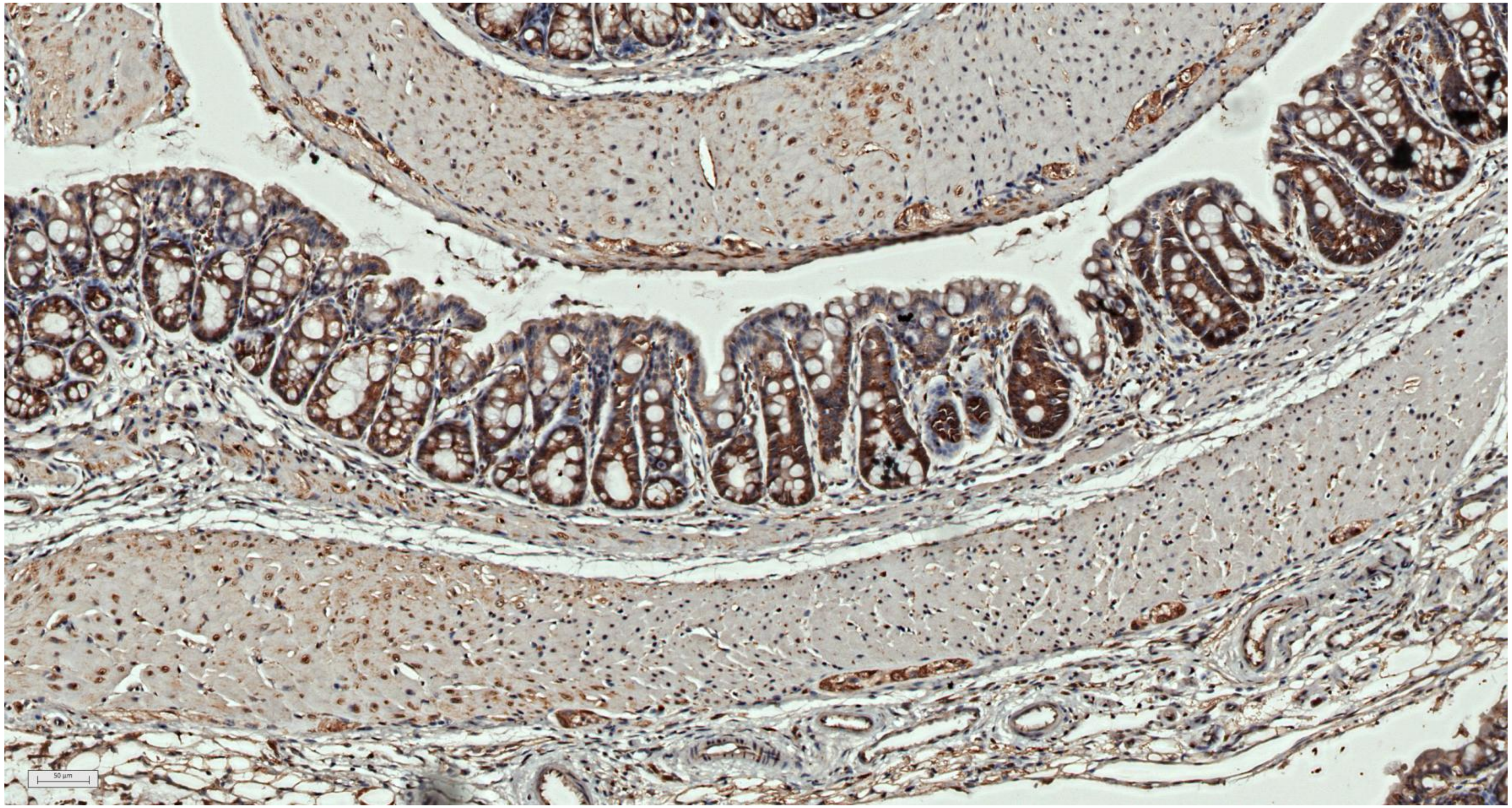

Fig. 5e (2.1)

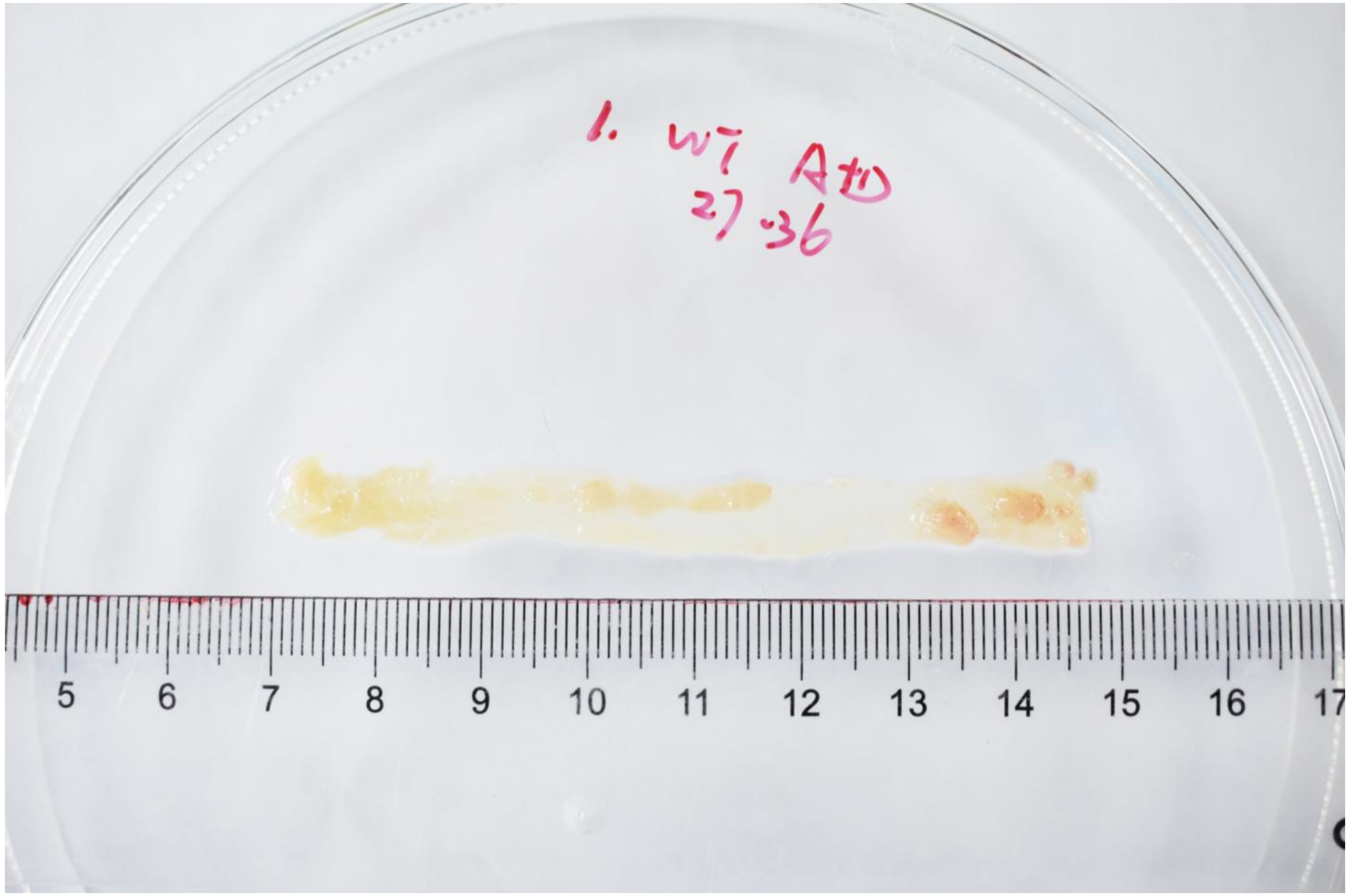

Fig. 4h (3.0)

Original image

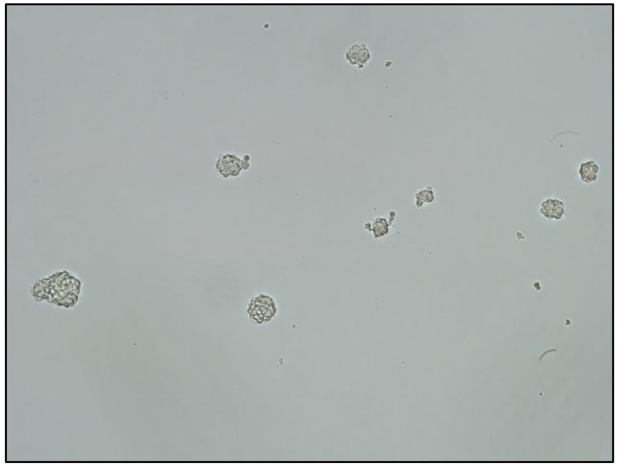

Fig. 6f (4.0)

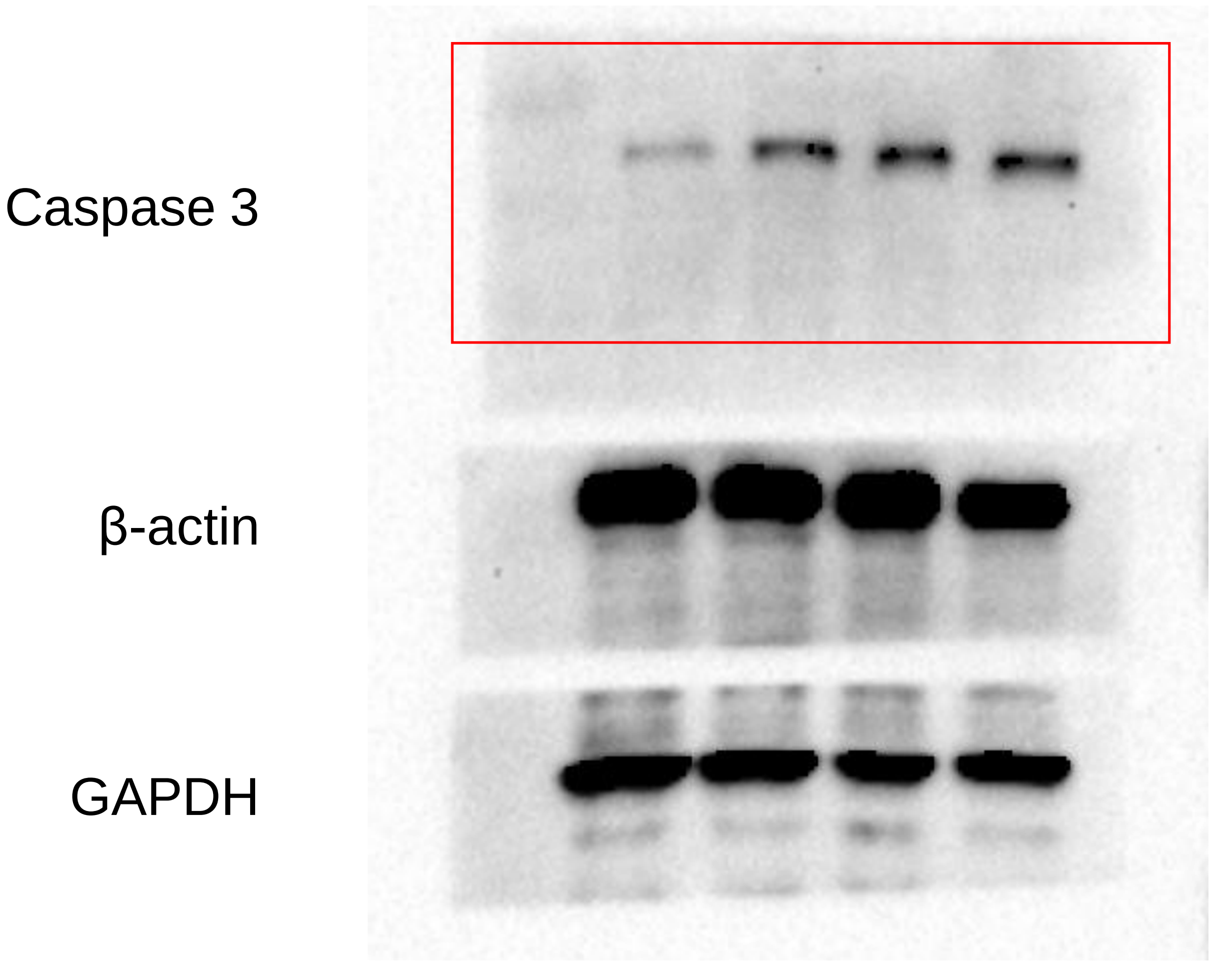

Fig. 8h (5.2-5.4)

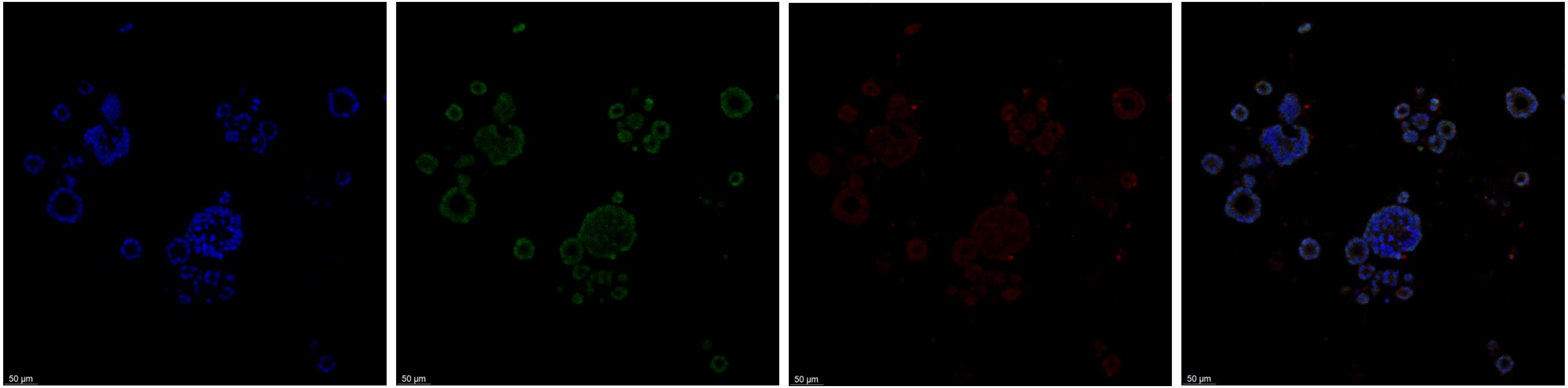

Fig. S3 (7.0)

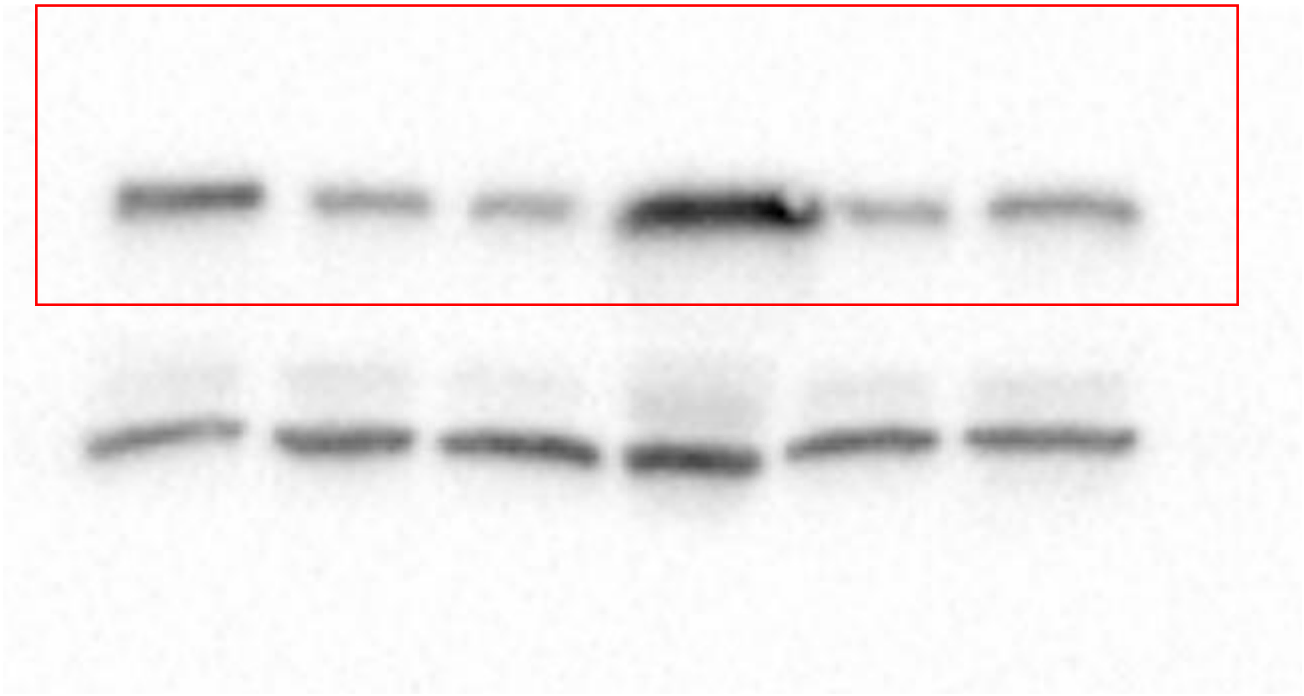

- 5.0, 5.1, and 6.0 depict tissue microarray (TMA) sections that exhibit high similarity owing to consistent immunohistochemical staining across adjacent serial sections. Nonetheless, these are distinct images derived from consecutive sectioning.
